# Supplementary material for: Spatial patterns of mosquito communities and monkey malaria vectors in a tropical riverine forest in Sabah, Malaysian Borneo
Source: PLoS Negl Trop Dis. 2026 Nov 3;20(11):e0014849. doi: 10.1371/journal.pntd.0014849 (PMC13759066; doi:10.1371/journal.pntd.0014849)
Supplement: Supplementary file 2 [file pntd.0014849.s007.pdf]

Comments to the authors

This study addresses a relevant question: how fine-scale spatial structuring of mosquito vectors along a river-forest gradient shapes opportunities for *P. knowlesi* transmission in Malaysian Borneo. The sampling design (two years, multiple distances and heights) is a strength, and so is the molecular confirmation of simian *Plasmodium* in *An. balabacensis*. I’ve summarized below a few suggestions for improvement.

1. Abstract

Major: Lines 50-51: five *Plasmodium* species were screened for (*P. knowlesi*, *P. cynomolgi*, *P. inui*, *P. coatneyi*, *P. fieldi*), but the species identity of the two positive *An. balabacensis* is never stated anywhere in the manuscript. This is not just a completeness issue: lines 448-450 state that the detection “suggests that transmission cycles may be active within the study area,” a claim that only supports the paper's *P. knowlesi* framing if the species is in fact *P. knowlesi*. Given that this motivates the whole study, it is the single most important result to specify precisely.

2. Introduction

Minor:

a. Lines 99-101: “particularly” appears twice in the same sentence (“particularly as modified by anthropogenic land-use changes... particularly infections caused by *Plasmodium knowlesi*”). Consider varying the phrasing.

b. Lines 150-152: “few studies have simultaneously examined mosquito community structure, vertical stratification, and distance from aquatic habitats in a single framework” is an uncited gap statement, and it is the sentence the paper's novelty claim rests on. Don't leave the gap unreferenced.

3. Methods

Study site

Major: Lines 206-208 note traps couldn't always be set at all six positions due to flooding, causing “slight variation” in sample numbers. Can you give the actual numbers achieved out of the 44x6 possible trap-nights, and state whether the missing nights were evenly distributed across Location-Height combinations?

Minor: The six trap locations sit 50 m off the central transect line to avoid disturbance from foot traffic. Were GPS coordinates recorded for each trap, or only nominal distance or height labels? Worth stating for reproducibility.

Statistical analyses

Minor:

a. The supplementary microclimate model was fit on “the same subset of data.” Please state how many observations were dropped for missing logger readings, and whether those nights overlap with the flooding-related missing data described under Study site.

b. “LOESS” (line 238) has never been defined. Please define it at first mention for readers outside biostatistics.

c. Lines 254-256 and 263: the *emmeans* package and R itself need references just as *glmmTMB* package.

4. Results

Overall

Major: One single trap (500 m, Tree) recorded 400 individuals in a single night, an extreme outlier in a design with only one trap per distance-height combination. Do the Location x Height effects in the GLMM hold up with that one observation removed? A meaningful share of the 500 m x Tree interaction could be driven by this single night.

Minor:

a. Lines 266-267: only the range (0-400) and total (9,928) are reported for mosquito abundance per sample. Please also report mean and SD (or median and IQR) given the data are zero-inflated. This would also help examine how extreme the 400-individual outlier mentioned above actually is relative to a typical sample.

b. Only one *Aedes* female was recorded across the whole study. CO2/light traps run overnight (17:00-07:30) and would miss day-biting *Aedes*. Worth a sentence noting the trap timing likely undersamples diurnal taxa, so genus-level conclusions about *Aedes* shouldn't be drawn from this dataset.

c. Lines 276-278: this closing sentence claims *Culex* dominance was “consistent... across all sampling conditions,” but no figure or table breaks down genus composition by distance, height, or time; the only support given is the single aggregate figure (91.4%) for the whole two-year dataset. Either soften “consistent across all sampling conditions” to reflect that this is an aggregate proportion, or report the disaggregated breakdown if it exists.

d. Lines 278-280: this restating of why analyses are restricted to females repeats what's already mentioned in Methods (lines 230-233). Consider cutting it.

Effects of spatial factors on overall mosquito abundance

Minor:

a. Table 1 shows the 250 m main effect at $P = 0.0487$, right at the conventional threshold, with five fixed-effect/interaction terms tested and no stated correction for multiple comparisons. Consider describing this particular result as borderline rather than at the same confidence as the height effect ($P = 0.0047$).

b. Table 1 shows the 250 m x Tree interaction is not significant ($P = 0.149$) while 500 m x Tree is ($P = 0.038$). The Discussion doesn't address why the height effect changes only at the farthest distance and not at the intermediate one. A sentence on the possible biological or sampling explanation would help.

Detection of monkey Plasmodium in Anopheles mosquitoes

Minor: Lines 318-319 (“these results confirm that infected *An. balabacensis* occurred within the trapping area”) reads more confidently than the Discussion's own caveat two pages later (lines 456-458), which correctly notes that whole-body PCR can't distinguish an infectious mosquito from one with a recent blood meal. I suggest you align the Results wording with the more careful phrasing already used in Discussion.

Effects of microclimatic conditions on mosquito abundance

Major: Lines 329-332: this conclusion frames spatial factors as explaining “substantial variation” versus microclimate's “comparatively little,” but no variance-explained metric (e.g., marginal R^2) is reported for either, in the main text or the Supplementary Tables. P-values and ΔAIC measure significance and relative fit, not magnitude of explained variation, so this comparison isn't actually supported by what's reported. The fair claim would be that no statistically significant microclimate effect was detected within the sampled range, not that spatial factors explained more. This framing is also repeated in the Discussion (lines 401-403).

5. Discussion

Vector community in the Borneo context

Major: Same parasite-identity gap as the Abstract comment: this paragraph (lines 362-375) discusses *An. balabacensis*'s general role as a vector of *Plasmodium knowlesi*, but never states which *Plasmodium* species the two field-detected positives actually carried. Worth stating the parasite species once, clearly, in whichever section the authors prefer, and referencing it elsewhere.

Environmental and spatial drivers of mosquito abundance

Minor: Lines 413-416: the zero-inflated model's high proportion of zero captures is interpreted as evidence of “structural absence” reflecting real spatial heterogeneity. Zero-inflation in a ZINB is a statistical fit decision, not by itself ecological proof of structural absence. Consider softening this claim or supporting it independently of the model specification.

Zoonotic risk and human-wildlife interfaces

Minor: Lines 492-501 state that the spatial findings are 'best interpreted as hypotheses about general patterns... rather than definitive tests,' since the design can't separate true distance/height effects from individual trap quirks. The very next paragraph (502-507) then says the results 'support the emerging consensus' on contact hotspots and are 'plausible foci for *P. knowlesi* spillover.' The findings were just described as hypotheses rather than definitive tests one sentence earlier, leaning on them to support a consensus claim two sentences later reads more confidently than the paper's own caveat allows. Consider tempering the language in 502-507 to match the hedge already given in 492-501."
