# Supplementary material for: Spatial patterns of mosquito communities and monkey malaria vectors in a tropical riverine forest in Sabah, Malaysian Borneo
Source: PLoS Negl Trop Dis. 2026 Nov 3;20(11):e0014849. doi: 10.1371/journal.pntd.0014849 (PMC13759066; doi:10.1371/journal.pntd.0014849)
Supplement: Supplementary file 3 [file pntd.0014849.s001.docx]

S1 Table . Mean nightly temperature and relative humidity recorded at each mosquito trap during the study period. Missing values (N/A) indicate logger failure or missing recordings.

| **Date** | **Distance** | **Stratum** | **Mean temperature (°C)** | **Mean relative humidity (%)** |
| --- | --- | --- | --- | --- |
| 2016-11-29 | 0 m | Ground | 27.1 | 90.7 |
| 2016-11-29 | 0 m | Tree | 26.8 | 92.7 |
| 2016-11-29 | 250 m | Ground | 27.0 | 90.8 |
| 2016-11-29 | 250 m | Tree | 26.8 | 90.9 |
| 2016-11-29 | 500 m | Ground | 26.8 | 91.2 |
| 2016-11-29 | 500 m | Tree | 26.9 | 89.6 |
| 2016-12-25 | 0 m | Ground | 27.2 | 91.8 |
| 2016-12-25 | 0 m | Tree | 27.1 | 98.7 |
| 2016-12-25 | 250 m | Ground | 27.0 | 96.2 |
| 2016-12-25 | 250 m | Tree | 26.9 | 94.6 |
| 2016-12-25 | 500 m | Ground | 26.9 | 94.1 |
| 2016-12-25 | 500 m | Tree | 26.8 | 91.5 |
| 2016-12-28 | 0 m | Ground | 27.6 | 89.7 |
| 2016-12-28 | 0 m | Tree | 27.4 | 95.3 |
| 2016-12-28 | 250 m | Ground | 27.6 | 91.3 |
| 2016-12-28 | 250 m | Tree | 27.4 | 91.0 |
| 2016-12-28 | 500 m | Ground | 27.4 | 91.3 |
| 2016-12-28 | 500 m | Tree | 27.3 | 87.9 |
| 2017-01-05 | 0 m | Ground | 27.4 | 87.2 |
| 2017-01-05 | 0 m | Tree | 27.0 | 92.2 |
| 2017-01-05 | 250 m | Ground | 27.3 | 87.3 |
| 2017-01-05 | 250 m | Tree | 27.1 | 86.5 |
| 2017-01-05 | 500 m | Ground | 27.0 | 89.4 |
| 2017-01-05 | 500 m | Tree | 27.1 | 85.3 |
| 2017-01-15 | 0 m | Ground | 26.8 | 89.3 |
| 2017-01-15 | 0 m | Tree | 26.5 | 93.2 |
| 2017-01-15 | 250 m | Ground | 26.7 | 89.9 |
| 2017-01-15 | 250 m | Tree | 26.3 | 89.0 |
| 2017-01-15 | 500 m | Ground | 26.5 | 91.3 |
| 2017-01-15 | 500 m | Tree | 26.5 | 87.3 |
| 2017-02-08 | 0 m | Ground | 26.6 | 86.2 |
| 2017-02-08 | 0 m | Tree | 26.2 | 88.9 |
| 2017-02-08 | 250 m | Ground | 26.3 | 88.5 |
| 2017-02-08 | 250 m | Tree | 25.9 | 89.7 |
| 2017-02-08 | 500 m | Ground | 25.9 | 89.6 |
| 2017-02-08 | 500 m | Tree | 25.9 | 87.7 |
| 2017-02-15 | 0 m | Ground | 25.4 | 86.2 |
| 2017-02-15 | 0 m | Tree | 25.2 | 89.4 |
| 2017-02-15 | 250 m | Ground | 25.4 | 86.7 |
| 2017-02-15 | 250 m | Tree | 25.4 | 85.6 |
| 2017-02-15 | 500 m | Ground | 25.2 | 88.8 |
| 2017-02-15 | 500 m | Tree | 25.3 | 84.1 |
| 2017-02-27 | 0 m | Ground | 26.0 | 99.9 |
| 2017-02-27 | 0 m | Tree | 25.7 | 100.0 |
| 2017-02-27 | 250 m | Ground | 25.8 | 100.0 |
| 2017-02-27 | 250 m | Tree | 23.5 | 98.8 |
| 2017-02-27 | 500 m | Ground | 25.7 | 100.0 |
| 2017-02-27 | 500 m | Tree | 25.5 | 92.5 |
| 2017-07-06 | 0 m | Ground | 27.5 | 89.6 |
| 2017-07-06 | 0 m | Tree | 27.4 | 92.2 |
| 2017-07-12 | 0 m | Ground | 26.4 | 91.9 |
| 2017-07-12 | 0 m | Tree | 26.4 | 94.1 |
| 2017-07-12 | 250 m | Ground | 26.4 | 89.7 |
| 2017-07-12 | 250 m | Tree | 26.1 | 89.3 |
| 2017-07-12 | 500 m | Ground | 26.2 | 94.1 |
| 2017-07-12 | 500 m | Tree | 26.2 | 89.0 |
| 2017-07-25 | 0 m | Ground | 29.0 | 82.5 |
| 2017-07-25 | 0 m | Tree | 29.0 | 86.8 |
| 2017-07-25 | 250 m | Ground | 29.2 | 80.2 |
| 2017-07-25 | 250 m | Tree | 29.1 | 81.6 |
| 2017-07-25 | 500 m | Ground | 28.9 | 85.9 |
| 2017-07-25 | 500 m | Tree | 28.8 | 80.8 |
| 2017-08-08 | 0 m | Ground | 27.9 | 85.4 |
| 2017-08-08 | 0 m | Tree | 28.2 | 88.2 |
| 2017-08-08 | 250 m | Ground | 28.5 | 82.9 |
| 2017-08-08 | 250 m | Tree | 27.9 | 84.5 |
| 2017-08-08 | 500 m | Ground | 28.0 | 87.8 |
| 2017-08-08 | 500 m | Tree | 28.0 | 83.2 |
| 2017-08-27 | 0 m | Ground | 28.0 | 85.1 |
| 2017-08-27 | 0 m | Tree | 27.6 | 89.7 |
| 2017-08-27 | 250 m | Ground | 27.9 | 85.4 |
| 2017-08-27 | 250 m | Tree | 27.6 | 64.1 |
| 2017-08-27 | 500 m | Ground | 27.7 | 88.5 |
| 2017-08-27 | 500 m | Tree | 27.5 | 85.6 |
| 2017-09-08 | 0 m | Ground | 29.1 | 80.9 |
| 2017-09-08 | 0 m | Tree | 28.8 | 86.1 |
| 2017-09-08 | 250 m | Ground | 28.9 | 81.9 |
| 2017-09-08 | 250 m | Tree | 28.6 | 54.1 |
| 2017-09-08 | 500 m | Ground | 28.6 | 85.1 |
| 2017-09-08 | 500 m | Tree | 28.8 | 81.3 |
| 2017-09-27 | 0 m | Ground | 26.4 | 90.6 |
| 2017-09-27 | 0 m | Tree | 26.2 | 94.9 |
| 2017-09-27 | 250 m | Ground | 26.3 | 91.3 |
| 2017-09-27 | 250 m | Tree | 29.1 | 42.5 |
| 2017-09-27 | 500 m | Ground | 26.1 | 94.4 |
| 2017-09-27 | 500 m | Tree | 26.0 | 91.7 |
| 2017-10-06 | 0 m | Ground | 27.3 | 87.4 |
| 2017-10-06 | 0 m | Tree | 27.0 | 92.1 |
| 2017-10-06 | 250 m | Ground | 27.2 | 86.9 |
| 2017-10-06 | 250 m | Tree | 28.1 | 46.9 |
| 2017-10-06 | 500 m | Ground | 27.1 | 90.7 |
| 2017-10-06 | 500 m | Tree | 26.9 | 87.8 |
| 2017-10-15 | 0 m | Ground | 27.2 | 88.8 |
| 2017-10-15 | 0 m | Tree | 26.9 | 93.8 |
| 2017-10-27 | 0 m | Ground | 27.0 | 88.2 |
| 2017-10-27 | 0 m | Tree | 26.6 | 93.5 |
| 2017-10-27 | 250 m | Ground | 27.0 | 88.9 |
| 2017-10-27 | 250 m | Tree | 27.0 | 61.2 |
| 2017-10-27 | 500 m | Ground | 26.9 | 91.0 |
| 2017-10-27 | 500 m | Tree | 26.9 | 88.6 |
| 2017-11-07 | 0 m | Ground | N/A | N/A |
| 2017-11-07 | 0 m | Tree | 26.8 | 93.7 |
| 2017-11-07 | 250 m | Ground | 27.3 | 88.6 |
| 2017-11-07 | 250 m | Tree | 27.2 | 61.3 |
| 2017-11-07 | 500 m | Ground | 26.9 | 92.4 |
| 2017-11-07 | 500 m | Tree | 26.9 | 89.1 |
| 2017-12-07 | 0 m | Ground | N/A | N/A |
| 2017-12-07 | 0 m | Tree | 26.7 | 93.5 |
| 2017-12-07 | 250 m | Ground | 27.3 | 88.8 |
| 2017-12-07 | 250 m | Tree | 27.1 | 63.5 |
| 2017-12-07 | 500 m | Ground | 27.2 | 91.2 |
| 2017-12-07 | 500 m | Tree | 27.0 | 89.5 |
| 2017-12-24 | 0 m | Ground | N/A | N/A |
| 2017-12-24 | 0 m | Tree | N/A | N/A |
| 2017-12-24 | 250 m | Ground | 27.2 | 91.4 |
| 2017-12-24 | 250 m | Tree | 27.0 | 62.7 |
| 2017-12-24 | 500 m | Ground | 26.8 | 94.7 |
| 2017-12-24 | 500 m | Tree | 26.9 | 88.6 |
| 2018-01-07 | 0 m | Ground | N/A | N/A |
| 2018-01-07 | 0 m | Tree | N/A | N/A |
| 2018-01-17 | 0 m | Ground | N/A | N/A |
| 2018-01-17 | 0 m | Tree | N/A | N/A |
| 2018-01-30 | 0 m | Ground | N/A | N/A |
| 2018-01-30 | 0 m | Tree | N/A | N/A |
| 2018-02-07 | 0 m | Ground | N/A | N/A |
| 2018-02-07 | 0 m | Tree | N/A | N/A |
| 2018-02-07 | 250 m | Ground | 24.7 | 93.2 |
| 2018-02-07 | 250 m | Tree | 24.4 | 62.1 |
| 2018-02-07 | 500 m | Ground | 24.2 | 96.8 |
| 2018-02-07 | 500 m | Tree | 24.4 | 93.1 |
| 2018-02-14 | 0 m | Ground | N/A | N/A |
| 2018-02-14 | 0 m | Tree | 25.5 | 91.7 |
| 2018-02-14 | 250 m | Ground | 25.4 | 90.2 |
| 2018-02-14 | 250 m | Tree | 25.2 | 68.7 |
| 2018-02-14 | 500 m | Ground | 25.4 | 92.5 |
| 2018-02-14 | 500 m | Tree | 25.1 | 88.3 |
| 2018-03-08 | 0 m | Ground | 26.8 | 89.3 |
| 2018-03-08 | 0 m | Tree | 26.6 | 92.5 |
| 2018-03-08 | 250 m | Ground | 26.6 | 90.7 |
| 2018-03-08 | 250 m | Tree | 26.3 | 58.9 |
| 2018-03-08 | 500 m | Ground | 26.4 | 93.2 |
| 2018-03-08 | 500 m | Tree | 26.3 | 90.0 |
| 2018-04-15 | 0 m | Ground | 27.5 | 87.9 |
| 2018-04-15 | 0 m | Tree | 27.1 | 91.7 |
| 2018-04-15 | 250 m | Ground | 27.3 | 87.8 |
| 2018-04-15 | 250 m | Tree | 27.2 | 59.2 |
| 2018-04-15 | 500 m | Ground | 27.0 | 92.3 |
| 2018-04-15 | 500 m | Tree | 27.0 | 88.0 |
| 2018-04-27 | 0 m | Ground | 27.7 | 87.2 |
| 2018-04-27 | 0 m | Tree | 27.4 | 92.1 |
| 2018-04-27 | 250 m | Ground | 27.7 | 86.4 |
| 2018-04-27 | 250 m | Tree | 27.4 | 66.0 |
| 2018-04-27 | 500 m | Ground | 27.4 | 91.4 |
| 2018-04-27 | 500 m | Tree | 27.3 | 87.5 |
| 2018-05-16 | 0 m | Ground | 27.7 | 87.3 |
| 2018-05-16 | 0 m | Tree | 27.6 | 90.4 |
| 2018-05-16 | 250 m | Ground | 27.6 | 83.0 |
| 2018-05-16 | 250 m | Tree | 27.6 | 64.5 |
| 2018-05-16 | 500 m | Ground | 27.4 | 90.8 |
| 2018-05-16 | 500 m | Tree | 27.3 | 87.1 |
| 2018-06-07 | 0 m | Ground | 28.3 | 85.3 |
| 2018-06-07 | 0 m | Tree | 28.3 | 88.0 |
| 2018-06-07 | 250 m | Ground | 28.4 | 80.1 |
| 2018-06-07 | 250 m | Tree | 28.1 | 68.4 |
| 2018-06-07 | 500 m | Ground | 28.1 | 88.4 |
| 2018-06-07 | 500 m | Tree | 28.1 | 84.3 |
| 2018-06-18 | 0 m | Ground | 29.9 | 78.3 |
| 2018-06-18 | 0 m | Tree | 29.6 | 83.0 |
| 2018-06-18 | 250 m | Ground | 29.5 | 74.1 |
| 2018-06-18 | 250 m | Tree | 29.6 | 71.1 |
| 2018-06-18 | 500 m | Ground | 29.2 | 84.2 |
| 2018-06-18 | 500 m | Tree | 29.5 | 77.7 |
| 2018-06-27 | 0 m | Ground | 25.1 | 93.2 |
| 2018-06-27 | 0 m | Tree | 25.0 | 97.3 |
| 2018-06-27 | 250 m | Ground | 25.0 | 89.8 |
| 2018-06-27 | 250 m | Tree | 24.9 | 58.4 |
| 2018-06-27 | 500 m | Ground | 24.9 | 97.3 |
| 2018-06-27 | 500 m | Tree | 24.7 | 93.2 |
| 2018-07-03 | 0 m | Ground | 26.7 | 89.6 |
| 2018-07-03 | 0 m | Tree | 26.7 | 93.3 |
| 2018-07-03 | 250 m | Ground | 26.5 | 87.1 |
| 2018-07-03 | 250 m | Tree | 26.3 | 64.3 |
| 2018-07-03 | 500 m | Ground | 26.4 | 93.9 |
| 2018-07-03 | 500 m | Tree | 26.3 | 89.0 |
| 2018-07-15 | 0 m | Ground | 28.3 | 82.4 |
| 2018-07-15 | 0 m | Tree | 28.1 | 86.8 |
| 2018-07-15 | 250 m | Ground | 28.1 | 79.5 |
| 2018-07-15 | 250 m | Tree | 28.1 | 72.0 |
| 2018-07-15 | 500 m | Ground | 28.0 | 86.2 |
| 2018-07-15 | 500 m | Tree | 28.1 | 80.5 |
| 2018-07-27 | 0 m | Ground | 30.6 | 77.6 |
| 2018-07-27 | 0 m | Tree | 30.1 | 82.7 |
| 2018-07-27 | 250 m | Ground | 30.8 | 74.4 |
| 2018-07-27 | 250 m | Tree | 30.2 | 73.8 |
| 2018-07-27 | 500 m | Ground | 30.6 | 79.9 |
| 2018-07-27 | 500 m | Tree | 30.2 | 76.9 |
| 2018-08-07 | 0 m | Ground | 27.1 | 86.6 |
| 2018-08-07 | 0 m | Tree | 27.0 | 89.9 |
| 2018-08-07 | 250 m | Ground | 27.3 | 84.2 |
| 2018-08-07 | 250 m | Tree | 26.8 | 67.6 |
| 2018-08-07 | 500 m | Ground | 26.9 | 89.7 |
| 2018-08-07 | 500 m | Tree | 26.6 | 86.6 |
| 2018-08-17 | 0 m | Ground | 28.6 | 80.0 |
| 2018-08-17 | 0 m | Tree | 28.2 | 83.8 |
| 2018-08-17 | 250 m | Ground | 28.3 | 79.4 |
| 2018-08-17 | 250 m | Tree | 28.1 | 70.1 |
| 2018-08-17 | 500 m | Ground | 29.4 | 78.7 |
| 2018-08-17 | 500 m | Tree | 28.0 | 80.0 |
| 2018-08-26 | 0 m | Ground | 27.5 | 84.6 |
| 2018-08-26 | 0 m | Tree | 28.7 | 83.3 |
| 2018-08-26 | 250 m | Ground | 27.4 | 83.1 |
| 2018-08-26 | 250 m | Tree | 27.2 | 69.3 |
| 2018-08-26 | 500 m | Ground | 26.9 | 90.0 |
| 2018-08-26 | 500 m | Tree | 27.2 | 83.7 |
| 2018-09-09 | 0 m | Ground | 27.2 | 84.7 |
| 2018-09-09 | 0 m | Tree | 27.0 | 90.6 |
| 2018-09-09 | 250 m | Ground | 26.7 | 85.3 |
| 2018-09-09 | 250 m | Tree | 26.5 | 67.9 |
| 2018-09-09 | 500 m | Ground | 26.6 | 88.6 |
| 2018-09-09 | 500 m | Tree | 26.5 | 85.3 |
| 2018-09-21 | 0 m | Ground | 27.8 | 83.2 |
| 2018-09-21 | 0 m | Tree | 27.6 | 87.6 |
| 2018-09-21 | 250 m | Ground | 28.0 | 80.1 |
| 2018-09-21 | 250 m | Tree | 27.5 | 74.6 |
| 2018-09-21 | 500 m | Ground | 27.9 | 85.7 |
| 2018-09-21 | 500 m | Tree | 27.5 | 82.1 |
| 2018-10-07 | 0 m | Ground | 28.7 | 79.4 |
| 2018-10-07 | 0 m | Tree | 28.0 | 84.1 |
| 2018-10-07 | 250 m | Ground | 28.4 | 77.8 |
| 2018-10-07 | 250 m | Tree | 28.8 | 73.6 |
| 2018-10-07 | 500 m | Ground | 28.7 | 80.5 |
| 2018-10-07 | 500 m | Tree | 27.9 | 79.6 |
| 2018-10-18 | 0 m | Ground | 26.5 | 88.0 |
| 2018-10-18 | 0 m | Tree | 26.2 | 92.0 |
| 2018-10-18 | 250 m | Ground | 26.3 | 86.1 |
| 2018-10-18 | 250 m | Tree | 26.0 | 66.3 |
| 2018-10-18 | 500 m | Ground | 26.1 | 91.5 |
| 2018-10-18 | 500 m | Tree | 26.0 | 88.1 |
| 2018-10-30 | 0 m | Ground | 28.4 | 84.6 |
| 2018-10-30 | 0 m | Tree | 28.3 | 89.2 |
| 2018-10-30 | 250 m | Ground | 28.3 | 81.5 |
| 2018-10-30 | 250 m | Tree | 28.1 | 73.1 |
| 2018-10-30 | 500 m | Ground | 28.3 | 88.4 |
| 2018-10-30 | 500 m | Tree | 28.2 | 83.4 |
