# Supplementary material for: Spatial patterns of mosquito communities and monkey malaria vectors in a tropical riverine forest in Sabah, Malaysian Borneo
Source: PLoS Negl Trop Dis. 2026 Nov 3;20(11):e0014849. doi: 10.1371/journal.pntd.0014849 (PMC13759066; doi:10.1371/journal.pntd.0014849)
Supplement: Supplementary file 4 [file pntd.0014849.s002.docx]

S2 Table . **Effect sizes from the zero-inflated negative binomial GLMM examining the influence of distance from the river (location), trap height (height), and their interaction on total mosquito abundance.** Values represent exponentiated coefficients (exp(Estimate)), interpreted as multiplicative changes in expected mosquito counts relative to the reference category (0 m, ground). Shown are point estimates and corresponding 95% confidence intervals.

| Predictor | exp(Estimate) | 95% CI |
| --- | --- | --- |
| Location 250m | 0.74 | 0.55–1.00 |
| Location 500m | 0.85 | 0.63–1.14 |
| Height (tree) | 0.66 | 0.50–0.88 |
| 250m × tree | 1.36 | 0.89–2.08 |
| 500m × tree | 1.55 | 1.02–2.36 |
