# Supplementary material for: Spatial patterns of mosquito communities and monkey malaria vectors in a tropical riverine forest in Sabah, Malaysian Borneo
Source: PLoS Negl Trop Dis. 2026 Nov 3;20(11):e0014849. doi: 10.1371/journal.pntd.0014849 (PMC13759066; doi:10.1371/journal.pntd.0014849)
Supplement: Supplementary file 5 [file pntd.0014849.s003.docx]

S3 Table. Parameter estimates and exponentiated effect sizes from the zero-inflated negative binomial GLMM evaluating the influence of night-time temperature and humidity on mosquito abundance. The model included distance from the river (location), sampling height (height), and their interaction as fixed effects, and sampling date as a random intercept. Estimates are presented on the log scale, along with standard errors (SE), z-values, and p-values. Exponentiated coefficients (exp(Estimate)) and 95% confidence intervals represent multiplicative changes in expected mosquito counts relative to the reference category (0 m, Ground). Neither temperature nor humidity significantly predicted mosquito abundance, and inclusion of these covariates did not improve model performance relative to the spatial model.

| **Predictor** | **Estimate** | **SE** | **z-value** | **p-value** | **exp(Estimate)** | **95% CI** |
| --- | --- | --- | --- | --- | --- | --- |
| Night-time temperature | -0.09434 | 0.07117 | -1.325 | 0.185 | 0.91 | 0.79–1.05 |
| Night-time humidity | 0.00287 | 0.00898 | 0.319 | 0.75 | 1 | 0.98–1.02 |
| Location 250 m | -0.3094 | 0.15936 | -1.941 | 0.052 | 0.73 | 0.53–1.00 |
| Location 500 m | -0.22616 | 0.16327 | -1.385 | 0.166 | 0.8 | 0.58–1.12 |
| Height (tree) | -0.47612 | 0.16263 | -2.928 | 0.003 | 0.62 | 0.45–0.86 |
| 250 m × tree | 0.38996 | 0.28289 | 1.379 | 0.168 | 1.48 | 0.85–2.58 |
| 500 m × tree | 0.53134 | 0.23637 | 2.248 | 0.025 | 1.7 | 1.07–2.70 |
