# Supplementary material for: Spatial patterns of mosquito communities and monkey malaria vectors in a tropical riverine forest in Sabah, Malaysian Borneo
Source: PLoS Negl Trop Dis. 2026 Nov 3;20(11):e0014849. doi: 10.1371/journal.pntd.0014849 (PMC13759066; doi:10.1371/journal.pntd.0014849)
Supplement: Supplementary file 6 [file pntd.0014849.s004.docx]

S1 Fig. **Map of the study area along the Menanggul River, Sabah, Malaysia, showing the sampling transect and mosquito trap locations.**

Mosquito traps were established at three distances from the river edge (0, 250, and 500 m), with paired ground- and canopy-level traps at each location. The overview world map is adapted from BlankMap-World.svg (Wikimedia Commons, Public Domain), while the detailed study-area map and sampling transect were created by the authors using GPS survey data.
