# Supplementary material for: Spatial patterns of mosquito communities and monkey malaria vectors in a tropical riverine forest in Sabah, Malaysian Borneo
Source: PLoS Negl Trop Dis. 2026 Nov 3;20(11):e0014849. doi: 10.1371/journal.pntd.0014849 (PMC13759066; doi:10.1371/journal.pntd.0014849)
Supplement: Supplementary file 7 [file pntd.0014849.s009.docx]

EDITOR’S COMMENTS RE PNTD-D-26-00934R1 (MATSUDA *ET AL.*)

At the bottom of the first part of the previous “Decision” letter, it was stated: “There are (will be) some Editor’s comments … this editorial input will be made on the basis of a revised manuscript.” Following on from that explanation, see below:

1. The word “simian” appears more than about 20 times in the main text. Consider avoiding the use of “simian” and replacing it (check the supplementary information as well) with the noun adjunct “monkey”, as in the title of the manuscript (assuming the plasmodial species in the two kinds of apes mentioned are not also relevant to the subject). You could alternatively write “non-human primate” in front of “malaria” in some places. The reason for not using “simian” appears in the following publication: “Culleton R, McFadden GI. Time to drop the term “simian malaria parasites’. *Trends Parasitol.* 2024;40(2):100-1. PMID 38129281. <https://doi.org/10.1016/j.pt.2023.11.010>

2. Review the manuscript for places where you are including or excluding humans when the word “primate(s)” is used. Insert “non-human” in front of “primate” when what you mean is “non-human primate” specifically: probably in lines 56, 79, 130, 136, 143, 144, 180, 501, 508, 511, 522 and 547, for instance. Elsewhere, you might clearly be excluding humans when using only “primate(s)”, thinking of line 108 (“primates” should stay like that in line 108). What about in lines 547 and 577?

3. Throughout the manuscript, use square brackets for reference numbers, as opposed to round brackets, without spaces between reference numbers. For example, “(2, 8)” in line 461 will become “[2,8]”.

4. Following on from the previous comment: Where there is a reference number range in the text, an en dash must be used, not a hyphen. Thus, in line 92, for example, “(3-5)” must become “[3–5]”. Make this correction (replacement of hyphens by en dashes for reference number ranges and closing up of gaps where there are spaces between reference numbers) throughout the manuscript.

5. For page number ranges in the reference list, use an en dash throughout, not a hyphen. As an example, “241-61” appears in line 608. This must be replaced by “241–61”.

6. Please change the first sentence of the figure legends so that they are in BOLD type. “Figure” should be abbreviated as “Fig” with no full stop after it (this being the style currently used by the journal) but with a full stop after the figure number. Thus, for example, the following will then appear in line 732: “**Fig 3. Spatial and microclimatic patterns in total mosquito abundance**”.

7. There is a lack of uniformity in respect of upper versus lower case in the words “Tree” and “Ground” (if not in some other words as well). Please go through the whole manuscript and introduce uniformity. In some places, though, the use of lower case is obviously correct (appropriate). But in some other places collectively, there is inconsistency. This matter of detail needs to receive attention before the publication process can be moved forward.

8. A full stop (period) after “vs” (abbreviation for “versus”) is more common in American English than in British English. Because the “s” is the last letter of the full word “versus”, convention is that the “s” does not need a full stop after it like it would after “v” (thus, “v.”). In view of the fact that British English is used in this manuscript (e.g., “behaviour” as opposed to “behavior”), consider deleting the full stop behind “vs” in lines 295, 328, 356, 741 and 817. In line 160, however, you already have “vs” without the full stop.

9. Line (L) 135: Insert “at” in front of “times”, because the word “in” at the beginning of the line does not “work” for “times of day”.

10. L 165: Substitute “determine” for “evaluate”.

11. L 178: Check whether there is an extra space(s) after “24 °C”, requiring removal.

12. L 180: Check whether there is a space between “tailed” and the hyphen in front of it that needs to be closed up.

13. L 205 & L 206: Text here reads: “… temperature and humidity loggers were attached to each trap and recorded at hourly intervals.” Presumably this means: “… temperature and humidity were recorded at hourly intervals by means of loggers attached to each trap”. In other words, it isn’t the “loggers” *per se* that were “recorded” (but, rather, their readings). Alter the wording so that the meaning is correct. Another was of dealing with this could be to write: “… temperature and humidity loggers that recorded at hourly intervals were attached to each trap.”

14. L 235: Think about whether “for” can be substituted for “targeting”, instead of repeating “targeting”, which also appears in line 233.

15. L 236 & L 237: In the main text, “*Plasmodium knowlesi*” appeared in full in line 101. Therefore, abbreviate it as “*P. knowlesi*” here. Also, tidy up the listing of the species names here by arranging them alphabetically. Check elsewhere in the manuscript for the same thing (although it is referred to below in comments no. 18 and no. 19).

16. L 288: “was” needs to be changed to “were” because “*Anopheles* spp.” is plural. But if you were to delete the “spp.” and write “*Anopheles*” only, as you have done in other places, then “was” will be correct.

17. L 327, L 329 & L 335: Commas appear before the percentage in the first two lines, but there is a semicolon in line 335. Introduce uniformity into the manuscript in this regard.

18. L 336 & L 337: List the *Anopheles* species names alphabetically, unless there is a reason for not doing so.

19. L 339: In this paragraph, species of *Plasmodium* are listed randomly in lines 342 and 343. Arrange the species names alphabetically, which would be uniformly neater, as later in the paragraph, where the names are in alphabetical order.

20. L 385: “in” monkey or “on” monkey?

21. L 390: In the main text, “*Plasmodium knowlesi*” appeared in full in line 101. Therefore, abbreviate it to “*P. knowlesi*” here.

22. L 420 & 421: The word “abundance” is used a lot in the manuscript. It is therefore suggested that for a change, “numbers found” be substituted here for “abundance observed”, which would in any case be much better wording for what is being described.

23. L 421: Insert “the” in front of “vertical”.

24. L 422: Alter “arise from” to “result from”.

25. L 452: Substitute “suggested” for “showed suggestive”.

26. L 480: Substitute “*An. balabacensis*” for “*Anopheles balabacensis*” because the full name previously appeared in the main text (in line 105).

27. L 494: Change “showed” to “was associated with”.

28. L 502: Substitute “often frequent” for “frequently use”.

29. L 502: Consider whether to insert a comma after “dusk”.

30. L 503 to L 505: The logic and wording could be improved by changing “… species, they may increase opportunities for host–vector contact” to “… species, the opportunities for host–vector contact may be increased”.

31. L 518: As a reminder of the manuscript-wide citing changes required, “(15-18, 21, 22)” in this line must be re-formatted as “[15–18,21,22]”.

32. L 551: It is correct to use a hyphen in the adjectival “high-risk”. But you have also used a hyphen in this line between “human” and “vector”; yet for similar NON-adjectives, an en dash sometimes appears elsewhere and sometimes not (e.g., in lines 468, 476, 496, 504 and 513). Go through the whole manuscript, keeping the hyphens for normal adjunct adjectives. However, make the obviously invented compound NON-adjectives uniform by using en dashes instead of hyphens, even though some of them might be used adjectivally. I will check this again in the re-revised manuscript to see if it “works”. Keep the two e**m** (as opposed to e**n**) dashes in lines 501 and 502.

33. L 553: Substitute “take place” for “occur”.

34. L 555: Think about whether insertion of the word “avoidance” after “exposure” would convey what you mean.

35. L 570: Add an “s” at the end of “Contribution” to make it plural.

36. L 582: Insert “the” in front of “Japan”.

37. L 606: At the end of line, alter “p.”, which is singular, to “pp.” (plural). Also do this in lines 672 and 685.

38. L 608: Correct the year of publication to “1964”.

39. L 625: “Malaria” appears with an upper case “M”. Use lower case (“m”).

40. L 627: For the first author (“Rahim”), give 4 initials, as in the original publication. Namely: “MAFA”.

41. L 634: For Wan-Sulaiman, substitute “W-Y” for “WY” as the initials of this author.

42. L 642: Behind “2018;8” add “:e47602”.

43. L 649: For “R H” in this line, substitute “Homathevi R”.

44. L 660: At the end of the line, delete the “e” in front of “86”. Incidentally, it is not there in the PDF of the paper, although PubMed does give it. Anyway, the “e” for the abbreviated last page number should be deleted.

45. L 661: Abbreviate the journal’s title as “Int J Primatol”.

46. L 662: Behind “Chua”, substitute “T-H” for “TH” (because that is what is in the original publication, and it should not be altered, therefore).

47. L 663: For “2022.”, substitute “2022;19(2):233–45.”

48. L 668: Change the first letter of “Ongoing” to a lower case “o”. This is as per the usual style of the journal following a colon in the title of an article. Do it in lines 656 and 678 as well.

49. L 668: In “2015-2017”, use an en dash, not a hyphen (“2015–2017”).

50. L 676: Leave the journal’s name as “Mammal Study”.

51. L 678: Abbreviate the name of the journal as “Mamm Biol”.

52. L 688: Change Bienvenu’s initials from “AL” to “A-L”.

53. L 693: Correct “2005;36:1-97” to “2005;36,Suppl2:1–97”.

54. L 694: Delete the duplicated Panthusiri name the second time it appears (PubMed is wrong). The correct authorship is shown on a PDF of the publication that can be accessed via Google Scholar.

55. L 695: Correct “2006;37:1-128” to “2006;37,Suppl2:1–128”.

56. L 699: Correct “2005;36:1-80” to “2005;36,Suppl1:1–80”.

57. L 702: Correct “2006;37:1-85” to “2006;37,Suppl1:1–85”.

58. L 703: Correct “Benthem KJv” to “van Benthem KJ”.

59. L 705: At the end of the reference, add: “:378–400.”.

60. L 707: Substitute “2025 ed.” for “ed2025”.

61. L 708: Substitute “R Development Core Team” for “R-Core-Development-Team”.

62. L 709: Insert “R” in front of “Foundation” so that this reads “R Foundation for Statistical Computing”.

63. L 711: The title of the journal should be “J Med Entomol”.

64. L 715: Behind “Wan-Sulaiman”, change “WY” to “W-Y”.

65. L 730: Insert a hyphen between “Mosquito” and “trapping”.

66. L 756: Correct “Figures” to “Figure” (delete the “s” at the end).

67. L 799: Insert a hyphen between “Mosquito” and “trapping” because of the adjectival usage there (as in line 730).

68. L 547: Either delete “infections” or change “malaria” to “plasmodial” (or “*Plasmodium*”). “Malaria infection” often appears in the literature. But “malaria” already means an infection, so to have “infection” after it is saying the same thing twice. Thus, the word “infections” in line 547 is superfluous, and “malaria” on its own is correct. By analogy, one does not for instance talk about “tuberculosis infection”. Only about “tuberculosis”.

MILES B. MARKUS (Editor)
