# Supplementary material for: Spatial patterns of mosquito communities and monkey malaria vectors in a tropical riverine forest in Sabah, Malaysian Borneo
Source: PLoS Negl Trop Dis. 2026 Nov 3;20(11):e0014849. doi: 10.1371/journal.pntd.0014849 (PMC13759066; doi:10.1371/journal.pntd.0014849)
Supplement: Supplementary file 8 [file pntd.0014849.s010.docx]

PLOS Neglected Tropical Diseases

Ref.:  Ms. No. PNTD-D-26-00934

Dear editors

Thank you for giving us the opportunity to revise our manuscript and respond to the Reviewers' comments.

Please find attached the revised manuscript with all revised text in grey shading. Our response to specific Reviewers’ comments is given at the bottom of this letter.

We thank you for your time and hope the manuscript now meets the requirements of *PLOS Neglected Tropical Diseases*.

Sincerely,

Ikki Matsuda on behalf of the authors

**Reviewer #1:**

In this manuscript, the authors discuss the relatively underexamined question of the ecology of monkey malaria vectors. This is reflective of its status as a neglected tropical disease and examining it is important due to the zoonotic nature of these parasites. Therefore, the work covered is significant and worth consideration. The authors have collected a large body of mosquitoes and examined the spatial structure of these populations. While I think this work is worthwhile, there are several concerns I have ranging from small to substantial. The introduction is largely suitable as it provided me a background to understand the environment and why the study needed to be performed. My major issue with the introduction is the ease of reading. There are extremely long sentences in the introduction that make it difficult to follow. I have highlighted a few points in the intro where splitting and reformatting the sentences that would make it more readable.

WE THANK THE REVIEWER FOR THE POSITIVE ASSESSMENT OF OUR STUDY AND FOR THE CONSTRUCTIVE COMMENTS. WE HAVE CAREFULLY REVISED THE MANUSCRIPT IN RESPONSE TO ALL OF THE POINTS RAISED. OUR DETAILED RESPONSES ARE PROVIDED BELOW.

| Reviewer’s comments | Our comments | Changes made in the text (lines refer to the revised version) |
| --- | --- | --- |
| The remaining issues I have are methodological. I will go from the minor to major issues. A minor issue I have (that just may be a preferential issue) is that I would have taken a subset of the An. balabacensis specimens and done some molecular work to confirm species. I understand that this is the dominant species in Sabah, but it would have been more robust to confirm that there was not the potential misidentification of a morphologically similar species such as An. dirus or latens, as it is not impossible that there were a few individuals present. However, that is me and is not necessarily an issue. | We agree that molecular confirmation of a subset of *Anopheles balabacensis* specimens would have provided an additional level of confidence in the species identification. In the present study, however, species identification was based on detailed morphological examination using established taxonomic keys by an experienced mosquito taxonomist. Because *An. balabacensis* is the predominant member of the Leucosphyrus Group reported from Sabah and the reviewer also notes that this is not necessarily a major issue, we have retained the original methodology. We acknowledge that molecular confirmation would be a useful addition in future studies. | None |
| Storing your samples in ethanol before PCR is. Ethanol storage causes problems with DNA degradation and residual ethanol can both reduce PCR efficacy. This would have been an issue with doing molecular characterisation. The next issue is that this could have affected how many parasites could have been identified. More importantly, identification of parasites by PCR presence is an issue. The presence of DNA does not indicate vector competence. It just indicates that an infected meal was taken. To incriminate the species as a vector, the presence of sporozoite would be needed, so either by ELISA or confirmation through RT-PCR. Therefore, the methodology does not confirm the individuals as vectors. This would have been controlled for by doing PCR on the Culex species. If absolutely no parasite DNA was found in the non-vectors, that would have strengthened (but not confirmed) the findings. However, it is possible that Culex females could have taken infected meals but not necessarily be vectors. | We agree that PCR detection of *Plasmodium* DNA from whole mosquito bodies does not, by itself, demonstrate vector competence, as it cannot distinguish parasites derived from infected blood meals from infective sporozoites in the salivary glands. We have therefore revised the Discussion to clarify this important limitation and to state explicitly that our findings should be interpreted as evidence of local *Plasmodium* circulation rather than direct confirmation of vector competence.  We also agree that screening non-vector mosquitoes, such as *Culex*, would have provided an additional line of evidence and could have strengthened the interpretation. However, such analyses were beyond the scope of the present study and remain an important direction for future research. | Changes made, L 485-492:  We detected *Plasmodium* DNA in two *An. balabacensis* individuals (one from 0 m ground trap, one from 0 m canopy trap). While this demonstrates that *Plasmodium*-positive *An. balabacensis* were present at the study site, the small number of positives precludes any inference about spatial patterns of infection risk or true prevalence. These detections should be interpreted as qualitative evidence of local *Plasmodium* circulation rather than quantitative estimates of infection pressure. Because PCR was performed on whole mosquito bodies, the detected *Plasmodium* DNA may have originated from either infected blood meals or infective sporozoites in the salivary glands. Therefore, these results should not be interpreted as direct evidence of vector competence. |
| The issue is that while these findings do not negate your findings, it does suggest a restructuring or reconsideration of your argument. It is notable that the authors do not have a dedicated limitations section. The simplest method to deal with these issues is to restructure the discussion with a limitation section to discuss these issues and how these additional methods/considerations would potentially alter the outcomes and conclusions. | We thank the reviewer for this helpful suggestion. Rather than introducing a separate “Limitations” section, we have strengthened the discussion of the study's limitations within the Discussion. In particular, we now more explicitly acknowledge the limitations associated with the lack of spatial replication, the use of whole-body PCR for *Plasmodium* detection, and the interpretation of the *Anopheles* observations as descriptive rather than statistically robust inferences. We believe that these revisions more clearly communicate the scope and limitations of our findings while maintaining the overall structure of the manuscript. | None – but please check the above improvement. |
| it would be useful to add a map of the collection sites as well as spreadsheet of the climate conditions. This can be added as supplementary data. | We added the map and climate data as the supplementary table (S1) and figure (S1) | See the newly added supplementary Table S1 and Figure S1. |
| The issue is that while these findings do not negate your findings, it does suggest a restructuring or reconsideration of your argument. It is notable that the authors do not have a dedicated limitations section. The simplest method to deal with these issues is to restructure the discussion with a limitation section to discuss these issues and how these additional methods/considerations would potentially alter the outcomes and conclusions. | We agree that the limitations of the study should be clearly articulated to place our findings in the appropriate context. Rather than introducing a separate "Limitations" section, we have strengthened the existing Discussion by explicitly introducing a paragraph that highlights the principal limitations of the study. This revised paragraph now begins with, “Several limitations should be considered when interpreting these findings,” and more clearly discusses how the lack of true spatial replication limits the generality of our conclusions and why the observed spatial patterns should be regarded as hypotheses requiring further validation through replicated studies. We believe that incorporating these points within the existing Discussion preserves the overall flow of the manuscript while providing a clearer and more balanced interpretation of our findings. | Additions made, 527-529:  Several limitations should be considered when interpreting these findings. Most importantly, our study lacks true replication at the trap level, with only one trap per distance × height combination. |

Reviewer #2:

This is a well-written and ecologically relevant study addressing an important question in zoonotic malaria ecology. The manuscript benefits from a substantial two-year sampling effort, clear ecological motivation, and integration of mosquito community data with broader questions of disease transmission risk. The major limitation is the lack of spatial replication and the small number of Anopheles mosquitoes collected. As a result, conclusions regarding simian malaria vectors and zoonotic transmission risk should be interpreted cautiously and framed as hypothesis-generating rather than definitive. Overall, I found the manuscript scientifically sound and suitable for publication after minor revision.

WE THANK THE REVIEWER FOR THE POSITIVE EVALUATION OF OUR MANUSCRIPT AND FOR THE CONSTRUCTIVE SUGGESTIONS. WE HAVE REVISED THE MANUSCRIPT ACCORDINGLY, AND OUR DETAILED RESPONSES TO EACH COMMENT ARE PROVIDED BELOW.

| Reviewer’s comments | Our comments | Changes made in the text (lines refer to the revised version) |
| --- | --- | --- |
| The objectives are clearly stated and the study addresses an important question regarding the spatial ecology of mosquito communities and potential simian malaria vectors in a riverine forest system. The sampling design is generally appropriate for the stated objectives and the two-year sampling period represents a strength of the study. The use of zero-inflated negative binomial GLMMs appears appropriate given the overdispersed count data and excess zeros.  The primary methodological limitation is the lack of spatial replication. Because only one trap was deployed for each distance x height combination, the effects of river distance and trap height cannot be fully separated from site-specific characteristics. The authors acknowledge this limitation and appropriately avoid formal statistical modeling of the sparse Anopheles dataset. However, I suggest emphasizing more explicitly that distance effects may be confounded with characteristics of the individual trap locations.  I did not identify major concerns regarding ethics or regulatory compliance. | We have revised the Discussion to emphasize more explicitly that the apparent effects of river distance may be partly confounded with characteristics of the individual trap locations and should therefore be interpreted cautiously. | Additions made, L 533-539:  Consequently, the apparent effects of river distance and vertical stratum should be interpreted cautiously, as they may partly reflect characteristics unique to the individual trap locations rather than these spatial factors themselves. Therefore, while our results document spatial variation at this site, they are best interpreted as hypotheses about general patterns in riverine forests rather than definitive tests. Future studies with replicated traps across multiple riverine systems will be necessary to establish the generality of these patterns. |
| The analyses presented are consistent with the stated objectives and analytical approach. Results are clearly presented and generally easy to follow.  One important consideration is that the statistical analyses are based primarily on total mosquito abundance, which was overwhelmingly dominated by Culex spp. In contrast, only 57 female Anopheles mosquitoes were collected and only two tested positive for Plasmodium DNA. So conclusions regarding simian malaria vectors are based largely on descriptive patterns rather than formal statistical inference. | We agree that the statistical analyses primarily reflect the spatial patterns of the overall mosquito assemblage, which was overwhelmingly dominated by *Culex* spp., whereas the limited number of *Anopheles* specimens precluded formal statistical analysis. As described in the Discussion, the *Anopheles* results are therefore interpreted as descriptive observations rather than statistically robust inferences. | None |
| The molecular screening results would benefit from additional detail. The methods indicate that positive samples were subjected to species-specific PCR assays for multiple simian malaria parasites, but the results only report detection of Plasmodium DNA in two Anopheles balabacensis individuals. Please clarify whether the infecting Plasmodium species could be identified and report those results. | We thank the reviewer for pointing this out. We agree that the Results should explicitly report the outcome of the species-specific PCR assays described in the Methods. We have therefore revised the Results to specify the *Plasmodium* species detected in the two positive *An. balabacensis* specimens. | Changes made, L 343-347  Species-specific PCR indicated mixed infections in both positive mosquitoes: one individual was positive for *P. coatneyi*, *P. fieldi*, and *P. inui*, whereas the other was positive for *P. coatneyi*, *P. cynomolgi*, *P. inui*, and *P. knowlesi*. These results demonstrate the occurrence of *Plasmodium* DNA-positive *An. balabacensis* within the trapping area. |
| The conclusions regarding mosquito community composition and broad spatial structuring of mosquito abundance are supported by the data.  However, some conclusions regarding river-edge habitats as zoonotic malaria hotspots should be presented somewhat more cautiously. The study demonstrates greater occurrence of Anopheles mosquitoes near the river at this site, but the low number of vector captures, the small number of infected specimens, and the lack of spatial replication limit the strength of inference regarding transmission risk. While the hypothesis that river-edge habitats facilitate increased vector-host-human overlap is reasonable, the present study does not directly measure host use, human activity, or transmission risk.  The authors appropriately discuss several important limitations and place their findings within the broader context of zoonotic malaria ecology. The manuscript contributes useful information relevant to understanding vector-host-human overlap and has clear public health relevance. | Thank you for this positive assessment. We agree that our data do not directly measure host use, human activity, or transmission risk, and that the limited number of *Anopheles* captures, the small number of *Plasmodium*-positive specimens, and the lack of spatial replication warrant a cautious interpretation of river-edge habitats as potential zoonotic malaria hotspots. To further address this point, we have revised the Discussion by (i) explicitly acknowledging that whole-body PCR cannot distinguish parasites derived from infected blood meals from infective sporozoites and therefore does not provide direct evidence of vector competence, and (ii) clarifying that the apparent effects of river distance should be interpreted cautiously because they may be partly reflect characteristics unique to the individual trap locations. We believe these revisions further strengthen the cautious interpretation of our findings. | Changes made, L 488-493:  These detections should be interpreted as qualitative evidence of local *Plasmodium* circulation rather than quantitative estimates of infection pressure. Because PCR was performed on whole mosquito bodies, the detected *Plasmodium* DNA may have originated from either infected blood meals or infective sporozoites in the salivary glands. Therefore, these results should not be interpreted as direct evidence of vector competence.  Additions made, L 532-536:  We cannot formally separate the effects of distance from the river or vertical stratum from site-specific characteristics of individual trap locations. Consequently, the apparent effects of river distance and vertical stratum should be interpreted cautiously, as they may partly reflect characteristics unique to the individual trap locations rather than these spatial factors themselves. |
| The conclusions regarding mosquito community composition and broad spatial structuring of mosquito abundance are supported by the data.  However, some conclusions regarding river-edge habitats as zoonotic malaria hotspots should be presented somewhat more cautiously. The study demonstrates greater occurrence of Anopheles mosquitoes near the river at this site, but the low number of vector captures, the small number of infected specimens, and the lack of spatial replication limit the strength of inference regarding transmission risk. While the hypothesis that river-edge habitats facilitate increased vector-host-human overlap is reasonable, the present study does not directly measure host use, human activity, or transmission risk.  The authors appropriately discuss several important limitations and place their findings within the broader context of zoonotic malaria ecology. The manuscript contributes useful information relevant to understanding vector-host-human overlap and has clear public health relevance. | We agree that our data do not directly measure host use, human activity, or transmission risk, and that conclusions regarding river-edge habitats should therefore be interpreted cautiously. To address this point, we have strengthened the Discussion in several places. Specifically, we (i) clarified that Plasmodium DNA detected by whole-body PCR does not constitute direct evidence of vector competence, (ii) explicitly acknowledged that the lack of spatial replication limits inference regarding river-distance effects, and (iii) introduced a dedicated paragraph highlighting the principal limitations of the study and emphasizing that our findings should be regarded as hypotheses requiring validation through replicated studies. We believe these revisions provide a more balanced and appropriately cautious interpretation of the potential role of river-edge habitats in zoonotic malaria transmission. | Changes made, L 488-493:  These detections should be interpreted as qualitative evidence of local *Plasmodium* circulation rather than quantitative estimates of infection pressure. Because PCR was performed on whole mosquito bodies, the detected *Plasmodium* DNA may have originated from either infected blood meals or infective sporozoites in the salivary glands. Therefore, these results should not be interpreted as direct evidence of vector competence.  Additions made, 527-536:  Several limitations should be considered when interpreting these findings. Most importantly, our study lacks true replication at the trap level, with only one trap per distance × height combination. This design choice, necessitated by logistical constraints associated with canopy access in a remote field site, means that our inferences about spatial patterns are strictly applicable to these specific sampling locations along a single transect. We cannot formally separate the effects of distance from the river or vertical stratum from site-specific characteristics of individual trap locations. Consequently, the apparent effects of river distance and vertical stratum should be interpreted cautiously, as they may partly reflect characteristics unique to the individual trap locations rather than these spatial factors themselves. |
| Consider reporting the proportion of trap nights with Anopheles detections to provide additional context regarding vector rarity. | Thank you for this suggestion. We agreed and added such information. | Additions made, 322-325:  Female *Anopheles* mosquitoes were detected only sporadically in the trapping dataset (57 individuals in total). They were detected in only 36 of the 244 trap-nights (14.8%), further illustrating their rarity within the sampled mosquito community. Nevertheless, their occurrence was clearly structured across space and vertical strata (Table 2). |
| Please provide a brief explanation for the relatively large number of mosquitoes that could not be identified to genus and discuss whether this may have influenced community composition estimates. | We added the explanation as suggested by the reviewer. | Additions made, L 287-293:  By contrast, *Anopheles* spp. was rare (153 individuals; 1.5%), and only a single *Aedes* female was recorded. Because trapping was conducted overnight (17:00–07:30), when most *Aedes* species are relatively inactive, these collections were not designed to effectively sample diurnal mosquitoes. The remaining 695 mosquitoes (7.0%) comprised other mosquito taxa and specimens that could not be identified to genus because many were damaged and lacked the diagnostic morphological characters required for reliable identification. |
| The Data Availability Statement indicates that all relevant data are contained within the manuscript and Supporting Information files. However, I was unable to locate the underlying trap-level mosquito abundance dataset or environmental measurements used in the analyses. Please ensure that the minimal dataset required to reproduce the reported analyses is available in the Supporting Information or an appropriate public repository. | We have now included the underlying trap-level mosquito abundance and environmental dataset used for the statistical analyses as Dataset.csv in the Supporting Information. The Data Availability Statement has been updated accordingly. | Please see the uploaded file “Dataset.csv”.  Changes made, L 590-591:  All relevant data underlying the findings of this study are provided within the manuscript and its Supporting Information files (Supplementary Dataset S1). |

Attached PDF 1:

This study addresses a relevant question: how fine-scale spatial structuring of mosquito vectors along a river-forest gradient shapes opportunities for P. knowlesi transmission in Malaysian Borneo. The sampling design (two years, multiple distances and heights) is a strength, and so is the molecular confirmation of simian Plasmodium in An. balabacensis. I’ve summarized below a few suggestions for improvement.

THANK YOU VERY MUCH FOR YOUR ALL VALUABLE COMMENTS. WE DID OUR BEST TO IMPROVE THE MANUSCRIPT BASED ON YOUR COMMENTS.

| Reviewer’s comments | Our comments | Changes made in the text (lines refer to the revised version) |
| --- | --- | --- |
| 1. Abstract  Major: Lines 50-51: five Plasmodium species were screened for (P. knowlesi, P. cynomolgi, P. inui, P. coatneyi, P. fieldi), but the species identity of the two positive An. balabacensis is never stated anywhere in the manuscript. This is not just a completeness issue: lines 448-450 state that the detection “suggests that transmission cycles may be active within the study area,” a claim that only supports the paper's P. knowlesi framing if the species is in fact P. knowlesi. Given that this motivates the whole study, it is the single most important result to specify precisely. | We have revised the Abstract, Results, and Discussion to report the results of the species-specific PCR assays. One *An. balabacensis* carried *P. coatneyi*, *P. fieldi*, and *P. inui*, whereas the other carried *P. coatneyi*, *P. cynomolgi*, *P. inui*, and *P. knowlesi*. The Abstract now also explicitly notes that *P. knowlesi* was detected among the simian *Plasmodium* species identified. | Changers made, L 51-52:  Molecular screening detected simian *Plasmodium* DNA in two *An. balabacensis* individuals, including *P. knowlesi*, together with other simian *Plasmodium* species.  Additions made, L 343-347:  Species-specific PCR indicated mixed infections in both positive mosquitoes: one individual was positive for *P. coatneyi*, *P. fieldi*, and *P. inui*, whereas the other was positive for *P. coatneyi*, *P. cynomolgi*, *P. inui*, and *P. knowlesi*. These results demonstrate the occurrence of *Plasmodium* DNA-positive *An. balabacensis* within the trapping area.  Changes made, L 480-484:  Given the established role of *Anopheles balabacensis* as a primary vector of simian malaria parasites in Sabah (13-15), the detection of *Plasmodium* DNA in two *An. balabacensis* individuals, including one positive for *P. knowlesi* together with other simian *Plasmodium*, species suggests that *Plasmodium* transmission may be occurring within the study area. |
| 2. Introduction  Minor:  a. Lines 99-101: “particularly” appears twice in the same sentence (“particularly as modified by anthropogenic land-use changes... particularly infections caused by Plasmodium knowlesi”).  Consider varying the phrasing. | We have revised the sentence to avoid repetition of “particularly” and improve readability. | Changes made, L 99-102:  In Southeast Asia, these ecological processes, especially those modified by anthropogenic land-use changes, are closely linked to the emergence of zoonotic malaria. In particular, infections caused by *Plasmodium knowlesi*, a simian malaria parasite, have become a major public health concern in Malaysian Borneo (10-12). |
| b. Lines 150-152: “few studies have simultaneously examined mosquito community structure, vertical stratification, and distance from aquatic habitats in a single framework” is an uncited gap statement, and it is the sentence the paper's novelty claim rests on. Don't leave the gap unreferenced. | We added several references as suggested. | Additions made, L 150-153:  Despite the potential importance of riverine habitats in shaping vector-host interactions, few studies have simultaneously examined mosquito community structure, vertical stratification, and distance from aquatic habitats in a single framework (14-16, 22, 26). |
| 3. Methods  Study site  Major: Lines 206-208 note traps couldn't always be set at all six positions due to flooding, causing “slight variation” in sample numbers. Can you give the actual numbers achieved out of the 44x6 possible trap-nights, and state whether the missing nights were evenly distributed across Location-Height combinations? | We have clarified the sampling effort by stating that 244 of the planned 264 trap-nights (92.4%) were successfully completed. We also specify that the missing trap-nights were caused by seasonal flooding that prevented access to the inland trapping locations (250 m and 500 m), whereas both river-edge (0 m) traps were sampled on all trapping dates. | Additions made, L 191-196:  On each trapping date, we deployed up to six traps simultaneously. Mosquito trapping was conducted on 44 dates. Of the planned 264 trap-nights (44 dates × 6 trap positions), 244 trap collections (92.4%) were successfully completed. The remaining 20 trap-nights were lost because seasonal flooding prevented access to the inland trapping locations (250 m and 500 m), whereas both river-edge (0 m) traps were successfully sampled on all trapping dates. |
| Minor: The six trap locations sit 50 m off the central transect line to avoid disturbance from foot traffic. Were GPS coordinates recorded for each trap, or only nominal distance or height labels? Worth stating for reproducibility. | Thank you – we explained this in details. | Additions made, L 211-218:  To minimize disturbance associated with repeated human access along the transect route, traps were positioned approximately 50 m away from the central transect line. The six trap locations were permanent sampling stations established during long-term ecological studies at the site. The central transect had previously been surveyed using GPS, and the 0 m, 250 m, and 500 m positions were permanently marked in the field. Accordingly, the same tagged trees were used repeatedly throughout the present study rather than recording GPS coordinates for each sampling occasion. Traps were set between 17:00 and 17:30 and retrieved between 07:00 and 07:30 the following morning. |
| Statistical analyses  Minor:  a. The supplementary microclimate model was fit on “the same subset of data.” Please state how many observations were dropped for missing logger readings, and whether those nights overlap with the flooding-related missing data described under Study site. | We added the information suggested by the reviewer. | Additions made, L 272-277:  Environmental variables were derived from hourly logger data as nightly (17:00–07:00) means for each Location-Height combination and merged with trap counts; the nightly temperature and relative humidity data used in the analyses are provided in Table S1. Complete environmental records were available for 231 of the 244 trap collections, and the supplementary model including temperature and humidity was therefore fitted to this subset of the data. |
| b. “LOESS” (line 238) has never been defined. Please define it at first mention for readers outside biostatistics. | We added the information. | Additions made, L 247-249:  Temporal patterns were displayed using scatterplots with locally estimated scatterplot smoothing (LOESS), faceted by distance from the river (0 m, 250 m, 500 m) and sampling height (Ground or Tree). |
| c. Lines 254-256 and 263: the emmeans package and R itself need references just as glmmTMB package. | We added the references. | Additions made, L 268-270:  Estimated marginal means (EMMs) and corresponding confidence intervals were obtained from the ZINB GLMM using the *emmeans* package (40) to aid interpretation of model coefficients.  Additions made, L 280-281:  All analyses were conducted in R version 4.4.1 (41). |
| 4. Results  Overall  Major: One single trap (500 m, Tree) recorded 400 individuals in a single night, an extreme outlier in a design with only one trap per distance-height combination. Do the Location x Height effects in the GLMM hold up with that one observation removed? A meaningful share of the 500 m x Tree interaction could be driven by this single night. | Thank you for this valuable suggestion. We conducted a sensitivity analysis excluding the single trap collection containing 400 mosquitoes. While the main effects of river distance and sampling height remained qualitatively unchanged, the interaction between river distance and sampling height was no longer statistically significant. We have added these results to both the Results and Discussion and now interpret the interaction more cautiously, while retaining the original analysis because the observation represents genuine biological variation. We have also added a brief description of this sensitivity analysis to the Methods section to improve the transparency of the analytical workflow. | Additions made, L 262-266:  This approach is appropriate for designs without spatial replication at the trap level. To evaluate the robustness of the primary model to the influence of a single exceptionally large mosquito capture, we also conducted a sensitivity analysis excluding the trap collection with the highest mosquito count (400 individuals).  Additions made, L 311-316:  In addition, the interaction between 500 m and tree height was significant (β = 0.44, SE = 0.21, P = 0.038), indicating that vertical differences in mosquito abundance varied across the river-distance gradient. However, sensitivity analysis excluding the single trap collection with the highest mosquito count (400 individuals) indicated that this interaction was no longer statistically significant (β = 0.29, SE = 0.20, P = 0.142), whereas the main effects of river distance and sampling height remained qualitatively unchanged.  Additions made, L :438-443:  To evaluate whether this single exceptionally large capture disproportionately influenced the GLMM results, we conducted a sensitivity analysis excluding this observation. The main effects of river distance and sampling height remained qualitatively unchanged, whereas the interaction between river distance and sampling height was no longer statistically significant. These findings indicate that the main spatial patterns were robust, whereas evidence for the interaction should be interpreted cautiously at this point. |
| Minor:  a. Lines 266-267: only the range (0-400) and total (9,928) are reported for mosquito abundance per sample. Please also report mean and SD (or median and IQR) given the data are zero-inflated. This would also help examine how extreme the 400-individual outlier mentioned above actually is relative to a typical sample. | We have added the median and interquartile range (IQR) of mosquito abundance per trap collection to better describe the distribution of the data and provide context for the single large capture event. | Additions made, L 284-285:  Total mosquito abundance per sample ranged from 0 to 400 individuals (median = 30, IQR = 14–55), with substantial variation both temporally and across sites (Figure 2). |
| b. Only one Aedes female was recorded across the whole study. CO2/light traps run overnight (17:00-07:30) and would miss day-biting Aedes. Worth a sentence noting the trap timing likely undersamples diurnal taxa, so genus-level conclusions about Aedes shouldn't be drawn from this dataset. | We have clarified that our overnight CO₂/light-trap sampling was not designed to effectively sample diurnal Aedes mosquitoes and that the very low number of Aedes collected should therefore be interpreted in the context of the sampling method. | Changes made, L 287-293:  By contrast, *Anopheles* spp. was rare (153 individuals; 1.5%), and only a single *Aedes* female was recorded. Because trapping was conducted overnight (17:00–07:30), when most *Aedes* species are relatively inactive, these collections were not designed to effectively sample diurnal mosquitoes. The remaining 695 mosquitoes (7.0%) comprised other mosquito taxa and specimens that could not be identified to genus because many were damaged and lacked the diagnostic morphological characters required for reliable identification. |
| c. Lines 276-278: this closing sentence claims Culex dominance was “consistent... across all sampling conditions,” but no figure or table breaks down genus composition by distance, height, or time; the only support given is the single aggregate figure (91.4%) for the whole two-year dataset. Either soften “consistent across all sampling conditions” to reflect that this is an aggregate proportion, or report the disaggregated breakdown if it exists. | We have revised the text to clarify that the reported dominance of *Culex* spp. refers to the overall mosquito assemblage rather than to all sampling conditions. | Changes made, L 299-300:  Overall, the dataset reveals strong temporal fluctuations in mosquito abundance, with *Culex* spp. accounting for over 90% of all mosquitoes collected. |
| d. Lines 278-280: this restating of why analyses are restricted to females repeats what's already mentioned in Methods (lines 230-233). Consider cutting it. | We have removed the repeated explanation as suggested by the reviewer. | Deleted |
| Effects of spatial factors on overall mosquito abundance  Minor:  a. Table 1 shows the 250 m main effect at P = 0.0487, right at the conventional threshold, with five fixed-effect/interaction terms tested and no stated correction for multiple comparisons. Consider describing this particular result as borderline rather than at the same confidence as the height effect (P = 0.0047). | We have slightly softened the description of the 250 m effect to reflect its marginal statistical support. | Changes made, L 305-307:  Mosquito counts tended to be lower at 250 m than at 0 m (β = –0.30, SE = 0.15, P = 0.049), corresponding to an approximately 26% decrease in expected abundance (Supplementary Table S2). |
| b. Table 1 shows the 250 m x Tree interaction is not significant (P = 0.149) while 500 m x Tree is (P = 0.038). The Discussion doesn't address why the height effect changes only at the farthest distance and not at the intermediate one. A sentence on the possible biological or sampling explanation would help. | Following your suggestion, we conducted a sensitivity analysis excluding the single exceptionally large mosquito capture. The main effects of river distance and sampling height remained qualitatively unchanged, whereas the interaction between river distance and sampling height was no longer statistically significant. We have incorporated these results into the Discussion and now interpret the interaction more cautiously. | Additions made, L 438-443:  To evaluate whether this single exceptionally large capture disproportionately influenced the GLMM results, we conducted a sensitivity analysis excluding this observation. The main effects of river distance and sampling height remained qualitatively unchanged, whereas the interaction between river distance and sampling height was no longer statistically significant. These findings indicate that the main spatial patterns were robust, whereas evidence for the interaction should be interpreted cautiously at this point. |
| Detection of monkey Plasmodium in Anopheles mosquitoes  Minor: Lines 318-319 (“these results confirm that infected An. balabacensis occurred within the trapping area”) reads more confidently than the Discussion's own caveat two pages later (lines 456-458), which correctly notes that whole-body PCR can't distinguish an infectious mosquito from one with a recent blood meal. I suggest you align the Results wording with the more careful phrasing already used in Discussion. | We have revised the Results to refer to the detection of *Plasmodium* DNA in *An. balabacensis*, thereby aligning the wording with the more cautious interpretation presented in the Discussion. | Changes made, L 346-347:  These results demonstrate the occurrence of *Plasmodium* DNA-positive *An. balabacensis* within the trapping area. |
| Effects of microclimatic conditions on mosquito abundance  Major: Lines 329-332: this conclusion frames spatial factors as explaining “substantial variation” versus microclimate's “comparatively little,” but no variance-explained metric (e.g., marginal R²) is reported for either, in the main text or the Supplementary Tables. P-values and ΔAIC measure significance and relative fit, not magnitude of explained variation, so this comparison isn't actually supported by what's reported. The fair claim would be that no statistically significant microclimate effect was detected within the sampled range, not that spatial factors explained more. This framing is also repeated in the Discussion (lines 401-403). | We agree that our analyses do not quantify the proportion of explained variation. We have therefore revised both the Results and Discussion to avoid comparing the relative explanatory power of spatial and microclimatic variables and instead state only that no statistically significant effects of short-term temperature or humidity were detected within the environmental conditions sampled. | Changes made, L 357-359:  These results indicate no statistically significant effects of night-time temperature or humidity on mosquito abundance within the range of environmental conditions sampled.  Changes made, L 428-431:  Although microclimate can influence mosquito development and survivorship in other contexts (6, 7), no statistically significant effects of short-term temperature or humidity were detected within the environmental range sampled in this study. |
| 5. Discussion  Vector community in the Borneo context  Major: Same parasite-identity gap as the Abstract comment: this paragraph (lines 362-375) discusses An. balabacensis's general role as a vector of Plasmodium knowlesi, but never states which Plasmodium species the two field-detected positives actually carried. Worth stating the parasite species once, clearly, in whichever section the authors prefer, and referencing it elsewhere. | We now explicitly state the *Plasmodium* species detected in the two positive *An. balabacensis* mosquitoes, including the detection of *P. knowlesi* together with other simian *Plasmodium* species, and refer to these results consistently throughout the manuscript. | Changes made, L 480-484:  Given the established role of *Anopheles balabacensis* as a primary vector of simian malaria parasites in Sabah (13-15), the detection of *Plasmodium* DNA in two *An. balabacensis* individuals, including one positive for *P. knowlesi* together with other simian *Plasmodium*, species suggests that *Plasmodium* transmission may be occurring within the study area. |
| Environmental and spatial drivers of mosquito abundance  Minor: Lines 413-416: the zero-inflated model's high proportion of zero captures is interpreted as evidence of “structural absence” reflecting real spatial heterogeneity. Zero-inflation in a ZINB is a statistical fit decision, not by itself ecological proof of structural absence. Consider softening this claim or supporting it independently of the model specification. | We agree that the zero-inflated model does not by itself demonstrate structural absence. We have therefore revised the Discussion to avoid this interpretation and instead state that the model is consistent with pronounced spatial heterogeneity in mosquito occurrence. | Changed made, L 446-449:  The use of zero-inflated models indicates that the observed mosquito counts included more zero captures than expected under a standard negative binomial model, consistent with pronounced spatial heterogeneity in mosquito occurrence. |
| Zoonotic risk and human-wildlife interfaces  Minor: Lines 492-501 state that the spatial findings are 'best interpreted as hypotheses about general patterns... rather than definitive tests,' since the design can't separate true distance/height effects from individual trap quirks. The very next paragraph (502-507) then says the results 'support the emerging consensus' on contact hotspots and are 'plausible foci for P. knowlesi spillover.' The findings were just described as hypotheses rather than definitive tests one sentence earlier, leaning on them to support a consensus claim two sentences later reads more confidently than the paper's own caveat allows. Consider tempering the language in 502-507 to match the hedge already given in 492-501." | We agree that this section should better reflect the exploratory nature of our findings. We have therefore revised the Discussion to state that our findings are consistent with the emerging consensus, rather than supporting it directly, and have further softened the wording regarding river-edge habitats as potential spillover foci. | Changes made, L 540-547:  Taken together, our findings are consistent with the emerging consensus that zoonotic malaria risk in Sabah is driven less by overall *Anopheles* abundance and more by localized “contact hotspots” where competent vectors, macaque and possibly langur reservoirs, and humans repeatedly co-occur in space and time. Riverine forest edges that attract both wildlife and people, particularly in fragmented and agriculturally modified landscapes, may therefore represent plausible foci for *P. knowlesi* spillover, despite low trap-based estimates of vector density. Future efforts should also focus on detecting malaria infections in the local primate population. |

Attached PDF 2:

| Reviewer’s comments | Our comments | Changes made in the text (lines refer to the revised version) |
| --- | --- | --- |
| L 102: There are several examples of where the reading could be simplified by making the sentences shorter. This is an example. Split here to simplify. | We have divided this sentence into two shorter sentences to improve readability. | Changes made, L 99-102:  In Southeast Asia, these ecological processes, especially those modified by anthropogenic land-use changes, are closely linked to the emergence of zoonotic malaria. In particular, infections caused by *Plasmodium knowlesi*, a simian malaria parasite, have become a major public health concern in Malaysian Borneo (10-12). |
| L 107: Start a new sentence here. | We improved the text. | Change made, L 104-109:  In Malaysian Borneo, several *Anopheles* species in the Leucosphyrus Group, especially *Anopheles balabacensis*, are recognized as primary vectors of simian malaria parasites (13-15). Human-landing studies have shown that *A. balabacensis* preferentially bites at ground level while still utilizing canopy strata, supporting its role as a potential bridge vector between arboreal primates and ground-dwelling humans in disturbed forest systems (9). |
| L 126: Split sentence here. | We improved the text. | Changes made, L 122-126:  Complementary landscape-scale analyses further show that forest fragmentation, proximity to plantation agriculture, and clearing around households are strongly associated with the distribution of *P. knowlesi* vector habitats and human exposure (20, 22). These findings indicate that anthropogenic land-use change reshapes vector ecology and creates new transmission interfaces. |
| L 172: A map would be useful here. | We have added a map of the study area as Supplementary Figure S1 to provide the geographic context of the sampling locations. | See figure S1. |
| L 217: Not the best way to store samples for DNA extraction. | Thank you for your comment. Although other preservation methods may provide advantages for DNA preservation, 95% ethanol is a widely used and practical method for field-based mosquito studies, and DNA quality was sufficient for the molecular analyses conducted here. We have therefore retained the original description. | None |
| L 270: That's because you collected at night and Aedes are day active. | We have added a sentence clarifying that the overnight sampling protocol was not designed to effectively sample diurnal *Aedes* mosquitoes and that the very low number of *Aedes* collected should therefore be interpreted in the context of the sampling method. | Additions made, L 287-293:  By contrast, *Anopheles* spp. was rare (153 individuals; 1.5%), and only a single *Aedes* female was recorded. Because trapping was conducted overnight (17:00–07:30), when most *Aedes* species are relatively inactive, these collections were not designed to effectively sample diurnal mosquitoes. The remaining 695 mosquitoes (7.0%) comprised other mosquito taxa and specimens that could not be identified to genus because many were damaged and lacked the diagnostic morphological characters required for reliable identification. |
