# Supplementary material for: Spatial patterns of mosquito communities and monkey malaria vectors in a tropical riverine forest in Sabah, Malaysian Borneo
Source: PLoS Negl Trop Dis. 2026 Nov 3;20(11):e0014849. doi: 10.1371/journal.pntd.0014849 (PMC13759066; doi:10.1371/journal.pntd.0014849)
Supplement: Supplementary file 9 [file pntd.0014849.s011.docx]

EDITOR’S COMMENTS – MATSUDA *ET AL*. – REVISION 2

1. As requested at the end of comment no. 48 in the editor’s previous list, use a lower case “a” after the colon in line 676 (reference no. 21) and a lower case “i” after the colon in line 705 (reference no. 30).

2. There should be en dashes (not hyphens) between page numbers in lines 622, 646 and 715.

3. Line (L) 93: At the end of the line, substitute “survival” for “survivorship”.

4. L 224: Consider inserting “level” between “species” and “using”.

5. L 321: Change “in” to “of”.

6. L 323: Alter “in only 36” to “during only 36”.

7. L 483: Correct the position of the comma in this line by moving it from behind “*Plasmodium*” to behind “species”.

8. L. 638: Substitute an en dash (as in the original publication) for the hyphen between the years.

9. L 686: Change “2022:233–45” to “2022;19(2): 233–45”. The omission concerned was pointed out in the previous list of editor’s comments but has been only partly corrected.

10. L 733: The alteration requested previously has not been done correctly. The name of the author concerned is not “Benthem KJ” (and previously, you had “Benthem KJv”). It is “**van Benthem KJ**”. If this is rectified at the proof stage, as opposed to before then, you should point out to the typesetters that the “v” in “van” is lower case (for this particular name), not upper case, strange as though this may seem.

11. L 738: For reference 41, you have omitted accurate information that was in Revision 1 of the manuscript, from Revision 2. The reference is now incomplete. That aside, there is a persisting mistake (which was one of the corrections in comments no. 61 and no. 62 in the editor’s previous list). Two words are the wrong way around. It is “Development Core”, not “Core Development”.

MILES B. MARKUS

(Academic Editor)
