# Supplementary material for: Spatial patterns of mosquito communities and monkey malaria vectors in a tropical riverine forest in Sabah, Malaysian Borneo
Source: PLoS Negl Trop Dis. 2026 Nov 3;20(11):e0014849. doi: 10.1371/journal.pntd.0014849 (PMC13759066; doi:10.1371/journal.pntd.0014849)
Supplement: Supplementary file 10 [file pntd.0014849.s012.docx]

Response to Editorial Comments

Manuscript ID: PNTD-D-26-00934R1

Title: *Spatial patterns of mosquito communities and monkey malaria vectors in a tropical riverine forest in Sabah, Malaysian Borneo*

Dear Editorial Office,

We sincerely thank the Editorial Office for the careful review of our manuscript and for the detailed editorial comments.

We have revised the manuscript thoroughly and have addressed all of the editorial comments to the best of our ability. These revisions include changes to terminology, formatting, figure legends, references, and other editorial issues throughout the manuscript.

For ease of review, we have uploaded both:

- a clean version of the revised manuscript, and
- a version with Track Changes enabled showing all revisions.

We appreciate your time and consideration and look forward to your further evaluation.

Sincerely,

Ikki Matsuda
(on behalf of all authors)
