# Supplementary material for: Spatial patterns of mosquito communities and monkey malaria vectors in a tropical riverine forest in Sabah, Malaysian Borneo
Source: PLoS Negl Trop Dis. 2026 Nov 3;20(11):e0014849. doi: 10.1371/journal.pntd.0014849 (PMC13759066; doi:10.1371/journal.pntd.0014849)
Supplement: Peer review history

# Peer review: Decision Letter 0

**DOI:** 10.1371/journal.pntd.0014849.r001

**Authors:** Amy Morrison, Miles Markus

29 Jun 2026

PNTD-D-26-00934

Spatial patterns of mosquito communities and monkey malaria vectors in a tropical riverine forest in Sabah, Malaysian Borneo

PLOS Neglected Tropical Diseases

Dear Prof. Matsuda,

Thank you for submitting your manuscript to PLOS Neglected Tropical Diseases. After careful consideration, we feel that it has merit but does not fully meet PLOS Neglected Tropical Diseases's publication criteria as it currently stands. Therefore, we invite you to submit a revised version of the manuscript that addresses the relatively straightforward points raised during the review process. Also, note the Academic Editor's explanation below.

Please submit your revised manuscript by 15 August 2026. If you will need more time than this to complete your revisions, please reply to this message or contact the journal office at plosntds@plos.org. When you're ready to submit your revision, log on to https://www.editorialmanager.com/pntd/ and select the 'Submissions Needing Revision' folder to locate your manuscript file.

Please include the following items when submitting your revised manuscript:

* A letter that responds to each point raised by the reviewers. You should upload that letter as a separate file labeled 'Response to Reviewers'. The file does not need to include responses to any formatting updates and technical items listed in the 'Journal Requirements' section below.

* A marked-up copy of your manuscript that highlights changes made to the original version. You should upload this as a separate file labeled 'Revised Manuscript with Track Changes'.

* An unmarked version of your revised paper without tracked changes. You should upload this as a separate file labeled 'Manuscript'.

If you would like to make changes to your financial disclosure, competing interests statement, or data availability statement, please make these updates within the submission form at the time of resubmission. Guidelines for resubmitting your figure files are available below the reviewer comments at the end of this letter.

As the corresponding author, your ORCID iD is verified in the submission system and will appear in the published article. PLOS supports the use of ORCID, and we encourage all coauthors to register for an ORCID iD and use it as well. Please encourage your coauthors to verify their ORCID iD within the submission system before final acceptance, as unverified ORCID iDs will not appear in the published article. Only the individual author can complete the verification step; PLOS staff cannot verify ORCID iDs on behalf of authors.

We look forward to receiving your revised manuscript.

Kind regards,

Miles B. Markus, Ph.D. (Imperial College London)

Academic Editor

PLOS Neglected Tropical Diseases

Amy Morrison

Section Editor

PLOS Neglected Tropical Diseases

Shaden Kamhawi

co-Editor-in-Chief

PLOS Neglected Tropical Diseases

orcid.org/0000-0003-4304-636XX

Paul Brindley

co-Editor-in-Chief

PLOS Neglected Tropical Diseases

orcid.org/0000-0003-1765-0002

**Additional Editor Comments:**

There are (will be) some Editor’s comments of a copyediting-type and related nature. But considering that you will be modifying parts of the text in response to what the reviewers have said, this editorial input will be made on the basis of a revised manuscript. In the meantime, you should react to the reviewers' reports. Some of the points have been raised by more than one reviewer, highlighting the need to address them.

**Journal Requirements:**

If the reviewer comments include a recommendation to cite specific previously published works, please review and evaluate these publications to determine whether they are relevant and should be cited. There is no requirement to cite these works unless the editor has indicated otherwise.

1) Please ensure that the CRediT author contributions listed for every co-author are completed accurately and in full.

At this stage, the following Authors/Authors require contributions: Ikki Matsuda, Benny Obrain Manin, Takaaki Yahiro, Primus Lambut, Joseph Tangah, Michael A. Huffman, Henry Bernard, Vijay Subbiah Kumar, and Tock Hing Chua. Please ensure that the full contributions of each author are acknowledged in the "Add/Edit/Remove Authors" section of our submission form.

The list of CRediT author contributions may be found here: https://journals.plos.org/plosntds/s/authorship#loc-author-contributions

2) Please upload all main figures as separate Figure files in .tif or .eps format. For more information about how to convert and format your figure files please see our guidelines:

https://journals.plos.org/plosntds/s/figures

3) We have noticed that you have uploaded Supporting Information files, but you have not included a list of legends. Please add a full list of legends for your Supporting Information files after the references list.

**Reviewers' Comments:**

Reviewer's Responses to Questions

**Key Review Criteria Required for Acceptance?**

As you describe the new analyses required for acceptance, please consider the following:

**Methods**

-Are the objectives of the study clearly articulated with a clear testable hypothesis stated?

-Is the study design appropriate to address the stated objectives?

-Is the population clearly described and appropriate for the hypothesis being tested?

-Is the sample size sufficient to ensure adequate power to address the hypothesis being tested?

-Were correct statistical analysis used to support conclusions?

-Are there concerns about ethical or regulatory requirements being met?

Reviewer #1: The remaining issues I have are methodological. I will go from the minor to major issues. A minor issue I have (that just may be a preferential issue) is that I would have taken a subset of the An. balabacensis specimens and done some molecular work to confirm species. I understand that this is the dominant species in Sabah, but it would have been more robust to confirm that there was not the potential misidentification of a morphologically similar species such as An. dirus or latens, as it is not impossible that there were a few individuals present. However, that is me and is not necessarily an issue.

Storing your samples in ethanol before PCR is. Ethanol storage causes problems with DNA degradation and residual ethanol can both reduce PCR efficacy. This would have been an issue with doing molecular characterisation. The next issue is that this could have affected how many parasites could have been identified. More importantly, identification of parasites by PCR presence is an issue. The presence of DNA does not indicate vector competence. It just indicates that an infected meal was taken. To incriminate the species as a vector, the presence of sporozoite would be needed, so either by ELISA or confirmation through RT-PCR. Therefore, the methodology does not confirm the individuals as vectors. This would have been controlled for by doing PCR on the Culex species. If absolutely no parasite DNA was found in the non-vectors, that would have strengthened (but not confirmed) the findings. However, it is possible that Culex females could have taken infected meals but not necessarily be vectors.

My biggest concern is something that was raised by the authors. The collection methods are biased towards Culex collections. Furthermore, the reason why there were not many Aedes collected is because you collected at night, and Aedes are day active (which is not mentioned by the authors). Animal baited collections or swarm collections may have altered the outcomes of this study.

Reviewer #2: The objectives are clearly stated and the study addresses an important question regarding the spatial ecology of mosquito communities and potential simian malaria vectors in a riverine forest system. The sampling design is generally appropriate for the stated objectives and the two-year sampling period represents a strength of the study. The use of zero-inflated negative binomial GLMMs appears appropriate given the overdispersed count data and excess zeros.

The primary methodological limitation is the lack of spatial replication. Because only one trap was deployed for each distance x height combination, the effects of river distance and trap height cannot be fully separated from site-specific characteristics. The authors acknowledge this limitation and appropriately avoid formal statistical modeling of the sparse Anopheles dataset. However, I suggest emphasizing more explicitly that distance effects may be confounded with characteristics of the individual trap locations.

I did not identify major concerns regarding ethics or regulatory compliance.

Reviewer #3: (No Response)

**********

**Results**

-Does the analysis presented match the analysis plan?

-Are the results clearly and completely presented?

-Are the figures (Tables, Images) of sufficient quality for clarity?

Reviewer #1: I am fine with the results section

Reviewer #2: The analyses presented are consistent with the stated objectives and analytical approach. Results are clearly presented and generally easy to follow.

One important consideration is that the statistical analyses are based primarily on total mosquito abundance, which was overwhelmingly dominated by Culex spp. In contrast, only 57 female Anopheles mosquitoes were collected and only two tested positive for Plasmodium DNA. So conclusions regarding simian malaria vectors are based largely on descriptive patterns rather than formal statistical inference.

The molecular screening results would benefit from additional detail. The methods indicate that positive samples were subjected to species-specific PCR assays for multiple simian malaria parasites, but the results only report detection of Plasmodium DNA in two Anopheles balabacensis individuals. Please clarify whether the infecting Plasmodium species could be identified and report those results.

Figures and tables appear to be of sufficient quality and effectively summarize the results.

Reviewer #3: (No Response)

**********

**Conclusions**

-Are the conclusions supported by the data presented?

-Are the limitations of analysis clearly described?

-Do the authors discuss how these data can be helpful to advance our understanding of the topic under study?

-Is public health relevance addressed?

Reviewer #1: The issue is that while these findings do not negate your findings, it does suggest a restructuring or reconsideration of your argument. It is notable that the authors do not have a dedicated limitations section. The simplest method to deal with these issues is to restructure the discussion with a limitation section to discuss these issues and how these additional methods/considerations would potentially alter the outcomes and conclusions.

Reviewer #2: The conclusions regarding mosquito community composition and broad spatial structuring of mosquito abundance are supported by the data.

However, some conclusions regarding river-edge habitats as zoonotic malaria hotspots should be presented somewhat more cautiously. The study demonstrates greater occurrence of Anopheles mosquitoes near the river at this site, but the low number of vector captures, the small number of infected specimens, and the lack of spatial replication limit the strength of inference regarding transmission risk. While the hypothesis that river-edge habitats facilitate increased vector-host-human overlap is reasonable, the present study does not directly measure host use, human activity, or transmission risk.

The authors appropriately discuss several important limitations and place their findings within the broader context of zoonotic malaria ecology. The manuscript contributes useful information relevant to understanding vector-host-human overlap and has clear public health relevance.

Reviewer #3: (No Response)

**********

**Editorial and Data Presentation Modifications?**

Use this section for editorial suggestions as well as relatively minor modifications of existing data that would enhance clarity. If the only modifications needed are minor and/or editorial, you may wish to recommend “Minor Revision” or “Accept”.

Reviewer #1: Minor modifications as per annotation on MS.

Minor issues: it would be useful to add a map of the collection sites as well as spreadsheet of the climate conditions. This can be added as supplementary data.

Reviewer #2: • Please clarify whether the two Plasmodium-positive Anopheles balabacensis specimens could be assigned to specific simian malaria species and report those results if available.

• Consider further emphasizing that river-distance effects cannot be fully separated from site-specific effects because only one trap was deployed for each distance x height combination.

• Consider tempering language referring to river-edge habitats as zoonotic malaria hotspots and clearly distinguishing between demonstrated findings and hypotheses regarding transmission risk.

• Consider reporting the proportion of trap nights with Anopheles detections to provide additional context regarding vector rarity.

• Please provide a brief explanation for the relatively large number of mosquitoes that could not be identified to genus and discuss whether this may have influenced community composition estimates.

• The Data Availability Statement indicates that all relevant data are contained within the manuscript and Supporting Information files. However, I was unable to locate the underlying trap-level mosquito abundance dataset or environmental measurements used in the analyses. Please ensure that the minimal dataset required to reproduce the reported analyses is available in the Supporting Information or an appropriate public repository.

Reviewer #3: (No Response)

**********

**Summary and General Comments**

Use this section to provide overall comments, discuss strengths/weaknesses of the study, novelty, significance, general execution and scholarship. You may also include additional comments for the author, including concerns about dual publication, research ethics, or publication ethics. If requesting major revision, please articulate the new experiments that are needed.

Reviewer #1: In this manuscript, the authors discuss the relatively underexamined question of the ecology of monkey malaria vectors. This is reflective of its status as a neglected tropical disease and examining it is important due to the zoonotic nature of these parasites. Therefore, the work covered is significant and worth consideration. The authors have collected a large body of mosquitoes and examined the spatial structure of these populations. While I think this work is worthwhile, there are several concerns I have ranging from small to substantial.

The introduction is largely suitable as it provided me a background to understand the environment and why the study needed to be performed. My major issue with the introduction is the ease of reading. There are extremely long sentences in the introduction that make it difficult to follow. I have highlighted a few points in the intro where splitting and reformatting the sentences that would make it more readable.

Reviewer #2: This is a well-written and ecologically relevant study addressing an important question in zoonotic malaria ecology. The manuscript benefits from a substantial two-year sampling effort, clear ecological motivation, and integration of mosquito community data with broader questions of disease transmission risk. The major limitation is the lack of spatial replication and the small number of Anopheles mosquitoes collected. As a result, conclusions regarding simian malaria vectors and zoonotic transmission risk should be interpreted cautiously and framed as hypothesis-generating rather than definitive. Overall, I found the manuscript scientifically sound and suitable for publication after minor revision.

Reviewer #3: (No Response)

**********

PLOS authors have the option to publish the peer review history of their article (what does this mean?). If published, this will include your full peer review and any attached files.

If you choose “no”, your identity will remain anonymous but your review may still be made public.

**Do you want your identity to be public for this peer review?**  For information about this choice, including consent withdrawal, please see our Privacy Policy.

Reviewer #1: No

Reviewer #2: No

Reviewer #3: No

[NOTE: If reviewer comments were submitted as an attachment file, they will be attached to this email and accessible via the submission site. Please log into your account, locate the manuscript record, and check for the action link "View Attachments". If this link does not appear, there are no attachment files.]

**Figure resubmission:**

While revising your submission, we strongly recommend that you use PLOS’s NAAS tool (https://ngplosjournals.pagemajik.ai/artanalysis) to test your figure files. NAAS can convert your figure files to the TIFF file type and meet basic requirements (such as print size, resolution), or provide you with a report on issues that do not meet our requirements and that NAAS cannot fix.

After uploading your figures to PLOS’s NAAS tool - https://ngplosjournals.pagemajik.ai/artanalysis, NAAS will process the files provided and display the results in the "Uploaded Files" section of the page as the processing is complete. If the uploaded figures meet our requirements (or NAAS is able to fix the files to meet our requirements), the figure will be marked as "fixed" above. If NAAS is unable to fix the files, a red "failed" label will appear above. When NAAS has confirmed that the figure files meet our requirements, please download the file via the download option, and include these NAAS processed figure files when submitting your revised manuscript.

**Reproducibility:**

To enhance the reproducibility of your results, we recommend that authors of applicable studies deposit laboratory protocols in protocols.io, where a protocol can be assigned its own identifier (DOI) such that it can be cited independently in the future. Additionally, PLOS ONE offers an option to publish peer-reviewed clinical study protocols. Read more information on sharing protocols at https://plos.org/protocols?utm_medium=editorial-email&utm_source=authorletters&utm_campaign=protocols

Attachment Submitted filename: PNTD-D-26-00934.pdf

Attachment Submitted filename: review_Matsuda_et_al.pdf

# Author comment: Author response to Decision Letter 1

**DOI:** 10.1371/journal.pntd.0014849.r002

29 Sep 2026

Attachment Submitted filename: IM_Mosq_RebuttalLet_R1.docx

# Editor report: Decision Letter 1

**DOI:** 10.1371/journal.pntd.0014849.r003

**Authors:** Amy Morrison, Miles Markus

13 Oct 2026

PNTD-D-26-00934R1

Spatial patterns of mosquito communities and monkey malaria vectors in a tropical riverine forest in Sabah, Malaysian BorneoPLOS Neglected Tropical DiseasesDear Dr Matsuda,Thank you for submitting your revised manuscript to PLOS Neglected Tropical Diseases, and for your clear explanation regarding the changes made.At the bottom of the first part of the previous "Decision" letter, it was explained: "There are (will be) some Editor's comments ... this editorial input will be made on the basis of a revised manuscript." Following on from that statement, see the comments below (which are duplicated in an attached file).  Please submit your re-revised manuscript by 3 December 2026. If you will need more time than this to complete your minor revisions, please reply to this message or contact the journal office at plosntds@plos.org. When you're ready to submit your revision, log on to https://www.editorialmanager.com/pntd/ and select the 'Submissions Needing Revision' folder to locate your manuscript file.Please include the following items when submitting your revised manuscript:* A letter that responds to each point raised by the editor. You should upload this letter as a separate file labeled 'Response to Editor'. This file does not need to include responses to any formatting updates and technical items listed in the 'Journal Requirements' section below.* A marked-up copy of your manuscript that highlights changes made to your first revision version. You should upload this as a separate file labeled 'Revised Manuscript with Track Changes'.* An unmarked version of your revised paper without tracked changes. You should upload this as a separate file labeled 'Manuscript'.If you would like to make changes to your financial disclosure, competing interests statement, or data availability statement, please make these updates within the submission form at the time of resubmission. Guidelines for resubmitting your figure files are available below the old (original) reviewer comments (that is, if any old ones are included) at the end of this letter.

As the corresponding author, your ORCID iD is verified in the submission system and will appear in the published article. PLOS supports the use of ORCID, and we encourage all coauthors to register for an ORCID iD and use it as well. Please encourage your coauthors to verify their ORCID iD within the submission system before final acceptance, as unverified ORCID iDs will not appear in the published article. Only the individual author can complete the verification step; PLOS staff cannot verify ORCID iDs on behalf of authors

We look forward to receiving your revised manuscript.Kind regards,Miles B. Markus, Ph.D. (Imperial College London)Academic EditorPLOS Neglected Tropical DiseasesAmy MorrisonSection EditorPLOS Neglected Tropical Diseases

Shaden Kamhawi

co-Editor-in-Chief

PLOS Neglected Tropical Diseases

orcid.org/0000-0003-4304-636XX

Paul Brindley

co-Editor-in-Chief

PLOS Neglected Tropical Diseases

orcid.org/0000-0003-1765-0002

**Additional Editor Comments:** EDITOR’S COMMENTS RE PNTD-D-26-00934R1 (MATSUDA ET AL.)

At the bottom of the first part of the previous “Decision” letter, it was stated: “There are (will be) some Editor’s comments … this editorial input will be made on the basis of a revised manuscript.” Following on from that explanation, see below:

1. The word “simian” appears more than about 20 times in the main text. Consider avoiding the use of “simian” and replacing it (check the supplementary information as well) with the noun adjunct “monkey”, as in the title of the manuscript (assuming the plasmodial species in the two kinds of apes mentioned are not also relevant to the subject). You could alternatively write “non-human primate” in front of “malaria” in some places. The reason for not using “simian” appears in the following publication: “Culleton R, McFadden GI. Time to drop the term “simian malaria parasites’. Trends Parasitol. 2024;40(2):100-1. PMID 38129281. https://doi.org/10.1016/j.pt.2023.11.010

2. Review the manuscript for places where you are including or excluding humans when the word “primate(s)” is used. Insert “non-human” in front of “primate” when what you mean is “non-human primate” specifically: probably in lines 56, 79, 130, 136, 143, 144, 180, 501, 508, 511, 522 and 547, for instance. Elsewhere, you might clearly be excluding humans when using only “primate(s)”, thinking of line 108 (“primates” should stay like that in line 108). What about in lines 547 and 577?

3. Throughout the manuscript, use square brackets for reference numbers, as opposed to round brackets, without spaces between reference numbers. For example, “(2, 8)” in line 461 will become “[2,8]”.

4. Following on from the previous comment: Where there is a reference number range in the text, an en dash must be used, not a hyphen. Thus, in line 92, for example, “(3-5)” must become “[3–5]”. Make this correction (replacement of hyphens by en dashes for reference number ranges and closing up of gaps where there are spaces between reference numbers) throughout the manuscript.

5. For page number ranges in the reference list, use an en dash throughout, not a hyphen. As an example, “241-61” appears in line 608. This must be replaced by “241–61”.

6. Please change the first sentence of the figure legends so that they are in BOLD type. “Figure” should be abbreviated as “Fig” with no full stop after it (this being the style currently used by the journal) but with a full stop after the figure number. Thus, for example, the following will then appear in line 732: “Fig 3. Spatial and microclimatic patterns in total mosquito abundance”.

7. There is a lack of uniformity in respect of upper versus lower case in the words “Tree” and “Ground” (if not in some other words as well). Please go through the whole manuscript and introduce uniformity. In some places, though, the use of lower case is obviously correct (appropriate). But in some other places collectively, there is inconsistency. This matter of detail needs to receive attention before the publication process can be moved forward.

8. A full stop (period) after “vs” (abbreviation for “versus”) is more common in American English than in British English. Because the “s” is the last letter of the full word “versus”, convention is that the “s” does not need a full stop after it like it would after “v” (thus, “v.”). In view of the fact that British English is used in this manuscript (e.g., “behaviour” as opposed to “behavior”), consider deleting the full stop behind “vs” in lines 295, 328, 356, 741 and 817. In line 160, however, you already have “vs” without the full stop.

9. Line (L) 135: Insert “at” in front of “times”, because the word “in” at the beginning of the line does not “work” for “times of day”.

10. L 165: Substitute “determine” for “evaluate”.

11. L 178: Check whether there is an extra space(s) after “24 °C”, requiring removal.

12. L 180: Check whether there is a space between “tailed” and the hyphen in front of it that needs to be closed up.

13. L 205 & L 206: Text here reads: “… temperature and humidity loggers were attached to each trap and recorded at hourly intervals.” Presumably this means: “… temperature and humidity were recorded at hourly intervals by means of loggers attached to each trap”. In other words, it isn’t the “loggers” per se that were “recorded” (but, rather, their readings). Alter the wording so that the meaning is correct. Another was of dealing with this could be to write: “… temperature and humidity loggers that recorded at hourly intervals were attached to each trap.”

14. L 235: Think about whether “for” can be substituted for “targeting”, instead of repeating “targeting”, which also appears in line 233.

15. L 236 & L 237: In the main text, “Plasmodium knowlesi” appeared in full in line 101. Therefore, abbreviate it as “P. knowlesi” here. Also, tidy up the listing of the species names here by arranging them alphabetically. Check elsewhere in the manuscript for the same thing (although it is referred to below in comments no. 18 and no. 19).

16. L 288: “was” needs to be changed to “were” because “Anopheles spp.” is plural. But if you were to delete the “spp.” and write “Anopheles” only, as you have done in other places, then “was” will be correct.

17. L 327, L 329 & L 335: Commas appear before the percentage in the first two lines, but there is a semicolon in line 335. Introduce uniformity into the manuscript in this regard.

18. L 336 & L 337: List the Anopheles species names alphabetically, unless there is a reason for not doing so.

19. L 339: In this paragraph, species of Plasmodium are listed randomly in lines 342 and 343. Arrange the species names alphabetically, which would be uniformly neater, as later in the paragraph, where the names are in alphabetical order.

20. L 385: “in” monkey or “on” monkey?

21. L 390: In the main text, “Plasmodium knowlesi” appeared in full in line 101. Therefore, abbreviate it to “P. knowlesi” here.

22. L 420 & 421: The word “abundance” is used a lot in the manuscript. It is therefore suggested that for a change, “numbers found” be substituted here for “abundance observed”, which would in any case be much better wording for what is being described.

23. L 421: Insert “the” in front of “vertical”.

24. L 422: Alter “arise from” to “result from”.

25. L 452: Substitute “suggested” for “showed suggestive”.

26. L 480: Substitute “An. balabacensis” for “Anopheles balabacensis” because the full name previously appeared in the main text (in line 105).

27. L 494: Change “showed” to “was associated with”.

28. L 502: Substitute “often frequent” for “frequently use”.

29. L 502: Consider whether to insert a comma after “dusk”.

30. L 503 to L 505: The logic and wording could be improved by changing “… species, they may increase opportunities for host–vector contact” to “… species, the opportunities for host–vector contact may be increased”.

31. L 518: As a reminder of the manuscript-wide citing changes required, “(15-18, 21, 22)” in this line must be re-formatted as “[15–18,21,22]”.

32. L 551: It is correct to use a hyphen in the adjectival “high-risk”. But you have also used a hyphen in this line between “human” and “vector”; yet for similar NON-adjectives, an en dash sometimes appears elsewhere and sometimes not (e.g., in lines 468, 476, 496, 504 and 513). Go through the whole manuscript, keeping the hyphens for normal adjunct adjectives. However, make the obviously invented compound NON-adjectives uniform by using en dashes instead of hyphens, even though some of them might be used adjectivally. I will check this again in the re-revised manuscript to see if it “works”. Keep the two em (as opposed to en) dashes in lines 501 and 502.

33. L 553: Substitute “take place” for “occur”.

34. L 555: Think about whether insertion of the word “avoidance” after “exposure” would convey what you mean.

35. L 570: Add an “s” at the end of “Contribution” to make it plural.

36. L 582: Insert “the” in front of “Japan”.

37. L 606: At the end of line, alter “p.”, which is singular, to “pp.” (plural). Also do this in lines 672 and 685.

38. L 608: Correct the year of publication to “1964”.

39. L 625: “Malaria” appears with an upper case “M”. Use lower case (“m”).

40. L 627: For the first author (“Rahim”), give 4 initials, as in the original publication. Namely: “MAFA”.

41. L 634: For Wan-Sulaiman, substitute “W-Y” for “WY” as the initials of this author.

42. L 642: Behind “2018;8” add “:e47602”.

43. L 649: For “R H” in this line, substitute “Homathevi R”.

44. L 660: At the end of the line, delete the “e” in front of “86”. Incidentally, it is not there in the PDF of the paper, although PubMed does give it. Anyway, the “e” for the abbreviated last page number should be deleted.

45. L 661: Abbreviate the journal’s title as “Int J Primatol”.

46. L 662: Behind “Chua”, substitute “T-H” for “TH” (because that is what is in the original publication, and it should not be altered, therefore).

47. L 663: For “2022.”, substitute “2022;19(2):233–45.”

48. L 668: Change the first letter of “Ongoing” to a lower case “o”. This is as per the usual style of the journal following a colon in the title of an article. Do it in lines 656 and 678 as well.

49. L 668: In “2015-2017”, use an en dash, not a hyphen (“2015–2017”).

50. L 676: Leave the journal’s name as “Mammal Study”.

51. L 678: Abbreviate the name of the journal as “Mamm Biol”.

52. L 688: Change Bienvenu’s initials from “AL” to “A-L”.

53. L 693: Correct “2005;36:1-97” to “2005;36,Suppl2:1–97”.

54. L 694: Delete the duplicated Panthusiri name the second time it appears (PubMed is wrong). The correct authorship is shown on a PDF of the publication that can be accessed via Google Scholar.

55. L 695: Correct “2006;37:1-128” to “2006;37,Suppl2:1–128”.

56. L 699: Correct “2005;36:1-80” to “2005;36,Suppl1:1–80”.

57. L 702: Correct “2006;37:1-85” to “2006;37,Suppl1:1–85”.

58. L 703: Correct “Benthem KJv” to “van Benthem KJ”.

59. L 705: At the end of the reference, add: “:378–400.”.

60. L 707: Substitute “2025 ed.” for “ed2025”.

61. L 708: Substitute “R Development Core Team” for “R-Core-Development-Team”.

62. L 709: Insert “R” in front of “Foundation” so that this reads “R Foundation for Statistical Computing”.

63. L 711: The title of the journal should be “J Med Entomol”.

64. L 715: Behind “Wan-Sulaiman”, change “WY” to “W-Y”.

65. L 730: Insert a hyphen between “Mosquito” and “trapping”.

66. L 756: Correct “Figures” to “Figure” (delete the “s” at the end).

67. L 799: Insert a hyphen between “Mosquito” and “trapping” because of the adjectival usage there (as in line 730).

68. L 547: Either delete “infections” or change “malaria” to “plasmodial” (or “Plasmodium”). “Malaria infection” often appears in the literature. But “malaria” already means an infection, so to have “infection” after it is saying the same thing twice. Thus, the word “infections” in line 547 is superfluous, and “malaria” on its own is correct. By analogy, one does not for instance talk about “tuberculosis infection”. Only about “tuberculosis”.

MILES B. MARKUS (Editor)**Journal Requirements:**

If the reviewer comments include a recommendation to cite specific previously published works, please review and evaluate these publications to determine whether they are relevant and should be cited. There is no requirement to cite these works unless the editor has indicated otherwise.

1) Please ensure that all Figure files have corresponding citations and legends within the manuscript. Currently, Figure Fig 3 in your submission file inventory does not have an in-text citation. If the figure is no longer to be included as part of the submission, please remove it from the file inventory.

2) Please amend your detailed Financial Disclosure statement. This is published with the article. It must therefore be completed in full sentences and contain the exact wording you wish to be published.

i) State what role the funders took in the study. If the funders had no role in your study, please state: "The funders had no role in study design, data collection and analysis, decision to publish, or preparation of the manuscript."

ii) If any authors received a salary from any of your funders, please state which authors and which funders..

If you did not receive any funding for this study, please simply state: u201cThe authors received no specific funding for this work.u201d

**Reviewers' comments:** [NOTE: If reviewer comments were submitted as an attachment file, they will be attached to this email and accessible via the submission site. Please log into your account, locate the manuscript record, and check for the action link "View Attachments". If this link does not appear, there are no attachment files.]**Figure resubmission:**  While revising your submission, we strongly recommend that you use PLOS’s NAAS tool (https://ngplosjournals.pagemajik.ai/artanalysis) to test your figure files. NAAS can convert your figure files to the TIFF file type and meet basic requirements (such as print size, resolution), or provide you with a report on issues that do not meet our requirements and that NAAS cannot fix.

After uploading your figures to PLOS’s NAAS tool - https://ngplosjournals.pagemajik.ai/artanalysis, NAAS will process the files provided and display the results in the "Uploaded Files" section of the page as the processing is complete. If the uploaded figures meet our requirements (or NAAS is able to fix the files to meet our requirements), the figure will be marked as "fixed" above. If NAAS is unable to fix the files, a red "failed" label will appear above. When NAAS has confirmed that the figure files meet our requirements, please download the file via the download option, and include these NAAS processed figure files when submitting your revised manuscript.**Reproducibility:** To enhance the reproducibility of your results, we recommend that authors of applicable studies deposit laboratory protocols in protocols.io, where a protocol can be assigned its own identifier (DOI) such that it can be cited independently in the future. Additionally, PLOS ONE offers an option to publish peer-reviewed clinical study protocols. Read more information on sharing protocols at https://plos.org/protocols?utm_medium=editorial-email&utm_source=authorletters&utm_campaign=protocols

Attachment Submitted filename: PNTD-D-26-00934R1 - Matsuda et al - Editor Comments.docx

# Author comment: Author response to Decision Letter 2

**DOI:** 10.1371/journal.pntd.0014849.r004

17 Oct 2026

Attachment Submitted filename: Response to Editorial Comments.docx

# Editor report: Decision Letter 2

**DOI:** 10.1371/journal.pntd.0014849.r005

**Authors:** Amy Morrison, Miles Markus

21 Oct 2026

Dear Prof. Matsuda,

We are pleased to inform you that your manuscript 'Spatial patterns of mosquito communities and monkey malaria vectors in a tropical riverine forest in Sabah, Malaysian Borneo' has been provisionally accepted for publication in PLOS Neglected Tropical Diseases.

**PLEASE NOTE THE EDITOR'S MINOR CORRECTIONS AND COMMENTS** below (duplicated in an attached file). These require attention at the proof stage, unless you get the opportunity to alter the manuscript before then.

Before your manuscript can be formally accepted you will need to complete some formatting changes, which you will receive in a follow up email. A member of our team will be in touch with a set of requests.

Please note that your manuscript will not be scheduled for publication until you have made the required changes, so a swift response would be appreciated.

IMPORTANT: The editorial review process is now complete. PLOS will only permit corrections to spelling, formatting or significant scientific errors from this point onwards. Requests for major changes, or any which affect the scientific understanding of your work, will cause delays to the publication date of your manuscript.

Should you, your institution's press office or the journal office choose to press release your paper, you will automatically be opted out of early publication. We ask that you notify us now if you or your institution is planning to press release the article. All press must be co-ordinated with PLOS.

Thank you again for supporting Open Access publishing; we are looking forward to publishing your work in PLOS Neglected Tropical Diseases.

Kind regards,

Miles B. Markus, Ph.D. (Imperial College London)

Academic Editor

PLOS Neglected Tropical Diseases

Amy Morrison

Section Editor

PLOS Neglected Tropical Diseases

Shaden Kamhawi

co-Editor-in-Chief

PLOS Neglected Tropical Diseases

orcid.org/0000-0003-4304-636XX

Paul Brindley

co-Editor-in-Chief

PLOS Neglected Tropical Diseases

orcid.org/0000-0003-1765-0002

***********************************************************

EDITOR’S COMMENTS – MATSUDA ET AL. – REVISION 2

1. As requested at the end of comment no. 48 in the editor’s previous list, use a lower case “a” after the colon in line 676 (reference no. 21) and a lower case “i” after the colon in line 705 (reference no. 30).

2. There should be en dashes (not hyphens) between page numbers in lines 622, 646 and 715.

3. Line (L) 93: At the end of the line, substitute “survival” for “survivorship”.

4. L 224: Consider inserting “level” between “species” and “using”.

5. L 321: Change “in” to “of”.

6. L 323: Alter “in only 36” to “during only 36”.

7. L 483: Correct the position of the comma in this line by moving it from behind “Plasmodium” to behind “species”.

8. L. 638: Substitute an en dash (as in the original publication) for the hyphen between the years.

9. L 686: Change “2022:233–45” to “2022;19(2): 233–45”. The omission concerned was pointed out in the previous list of editor’s comments but has been only partly corrected.

10. L 733: The alteration requested previously has not been done correctly. The name of the author concerned is not “Benthem KJ” (and previously, you had “Benthem KJv”). It is “van Benthem KJ”. If this is rectified at the proof stage, as opposed to before then, you should point out to the typesetters that the “v” in “van” is lower case (for this particular name), not upper case, strange as though this may seem.

11. L 738: For reference 41, you have omitted accurate information that was in Revision 1 of the manuscript, from Revision 2. The reference is now incomplete. That aside, there is a persisting mistake (which was one of the corrections in comments no. 61 and no. 62 in the editor’s previous list). Two words are the wrong way around. It is “Development Core”, not “Core Development”.

MILES B. MARKUS

(Academic Editor)

Attachment Submitted filename: Editor Comments on Matsuda et al - Revision 2.docx

# Editor report: Acceptance letter

**DOI:** 10.1371/journal.pntd.0014849.r006

**Authors:** Amy Morrison, Miles Markus

Dear Dr. Matsuda,

We are delighted to inform you that your manuscript, "Spatial patterns of mosquito communities and monkey malaria vectors in a tropical riverine forest in Sabah, Malaysian Borneo," has been formally accepted for publication in PLOS Neglected Tropical Diseases.

We have now passed your article onto the PLOS Production Department who will complete the rest of the publication process. All authors will receive a confirmation email upon publication.

The corresponding author will soon be receiving a typeset proof for review, to ensure errors have not been introduced during production. Please review the PDF proof of your manuscript carefully, as this is the last chance to correct any scientific or type-setting errors. Please note that major changes, or those which affect the scientific understanding of the work, will likely cause delays to the publication date of your manuscript. Note: Proofs for Front Matter articles (Editorial, Viewpoint, Symposium, Review, etc...) are generated on a different schedule and may not be made available as quickly.

Soon after your final files are uploaded, the early version of your manuscript will be published online unless you opted out of this process. The date of the early version will be your article's publication date. The final article will be published to the same URL, and all versions of the paper will be accessible to readers.

For Research Articles, you will receive an invoice from PLOS for your publication fee after your manuscript has reached the completed accept phase. If you receive an email requesting payment before acceptance or for any other service, this may be a phishing scheme. Learn how to identify phishing emails and protect your accounts at https://explore.plos.org/phishing.

Thank you again for supporting open-access publishing; we are looking forward to publishing your work in PLOS Neglected Tropical Diseases.

Best regards,

Shaden Kamhawi

co-Editor-in-Chief

PLOS Neglected Tropical Diseases

Paul Brindley

co-Editor-in-Chief

PLOS Neglected Tropical Diseases
